# A retrospective epidemiological analysis of human *Cryptosporidium* infection in China during the past three decades (1987-2018)

**DOI:** 10.1371/journal.pntd.0008146

**Published:** 2020-03-30

**Authors:** Aiqin Liu, Baiyan Gong, Xiaohua Liu, Yujuan Shen, Yanchen Wu, Weizhe Zhang, Jianping Cao

**Affiliations:** 1 Department of Parasitology, Harbin Medical University, Harbin, Heilongjiang, China; 2 National Institute of Parasitic Diseases, Chinese Center for Disease Control and Prevention; Chinese Center for Tropical Diseases Research; WHO Collaborating Center`for Tropical Diseases; National Center for International Research on Tropical Diseases, Ministry of Science and Technology; Key Laboratory of Parasite and Vector Biology, MOH; Shanghai, China; University of North Carolina at Chapel Hill, UNITED STATES

## Abstract

**Background:**

Cryptosporidiosis is an emerging infectious disease of public health significance worldwide. The burden of disease caused by *Cryptosporidium* varies between and within countries/areas. To have a comprehensive understanding of epidemiological status and characteristics of human *Cryptosporidium* infection in China since the first report in 1987, a retrospective epidemiological analysis was conducted by presenting differences in the prevalence of *Cryptosporidium* by province, year, population, living environment and season and possible transmission routes and risk factors as well as genetic characteristics of *Cryptosporidium* in humans.

**Methodology/Principal findings:**

A systematic search was conducted to obtain epidemiological papers of human *Cryptosporidium* infection/cryptosporidiosis from PubMed and Chinese databases. Finally, 164 papers were included in our analysis. At least 200,054 people from 27 provinces were involved in investigational studies of *Cryptosporidium*, with an average prevalence of 2.97%. The prevalence changed slightly over time. Variable prevalences were observed: 0.65–11.15% by province, 1.89–47.79% by population, 1.77–12.87% and 0–3.70% in rural and urban areas, respectively. The prevalence peak occurred in summer or autumn. Indirect person-to-person transmission was documented in one outbreak of cryptosporidiosis in a pediatric hospital. 263 *Cryptosporidium* isolates were obtained, and seven *Cryptosporidium* species were identified: *C*. *hominis* (48.3%), *C*. *andersoni* (22.43%), *C*. *parvum* (16.7%), *C*. *meleagridis* (8.36%), *C*. *felis* (3.04%), *C*. *canis* (0.76%) and *C*. *suis* (0.38%).

**Conclusions/Significances:**

This systematic review reflects current epidemiological status and characteristics of *Cryptosporidium* in humans in China. These data will be helpful to develop efficient control strategies to intervene with and prevent occurrence of human *Cryptosporidium* infection/cryptosporidiosis in China as well as have a reference effect to other countries. Further studies should focus on addressing a high frequency of *C*. *andersoni* in humans and a new challenge with respect to cryptosporidiosis with an increasing population of elderly people and patients with immunosuppressive diseases.

## Author summary

*Cryptosporidium* is a major cause of diarrheal disease in humans globally. Due to the lack of effective drug treatment and vaccine prevention against cryptosporidiosis, it is particularly important to develop efficient control strategies to intervene with and prevent *Cryptosporidium* infection in humans. The present review presented and analyzed epidemiological status and characteristics of *Cryptosporidium* infection in humans in China since the first report in 1987. To date, epidemiological investigations of *Cryptosporidium* infecion have been carried out in different populations in 27 provinces, autonomous regions, and municipalities. Average prevalence of *Cryptosporidium* was 2.97% (5,933/200,054). Like other infectious disease, due to poor sanitation conditions in rural areas, people living in rural areas had a significantly higher prevalence of *Cryptosporidium* (1.77–12.87%) than those living in urban areas (0–3.70%). Seven *Cryptosporidium* species were identified, including *C*. *hominis*, *C*. *andersoni*, *C*. *parvum*, *C*. *meleagridis*, *C*. *feli*s, *C*. *canis* and *C*. *suis*. This retrospective epidemiological analysis indicates wide geographical distribution of human *Cryptosporidium* infection/cryptosporidiosis in China.

## Introduction

*Cryptosporidium* species are common protozoan parasites that infect the epithelial cells of the intestinal tract of humans and a variety of animals worldwide. Diarrhea is the common clinical symptom of cryptosporidiosis in infected hosts, varying depending on their health status. In humans, immune-competent individuals usually experience self-limiting diarrhea; however, immune-compromised individuals, particularly those with HIV infection, often suffer from intractable diarrhea [1]. *Cryptosporidium* is defined as one of the second highest priority organisms/biological agents by the National Institutes of Health (NIH) of the USA and cryptosporidiosis is recognized as the major cause of diarrhea in patients with AIDS and life-threatening diarrhea has been reported in this population [1]. In the Global Burden of Disease Study (GBD) 2016, *Cryptosporidium* was the fifth leading cause of diarrheal mortality in children younger than five years, and 84.4% (48,300/57,200) of deaths from *Cryptosporidium* infection occurred in this age group [2].

Cryptosporidiosis is a highly prevalent and widespread disease documented in humans in over 90 countries on all continents except Antarctica [3]. The burden of disease from cryptosporidiosis varies substantially between and within countries/areas. The prevalence of *Cryptosporidium* in humans is reported to be 2.6–21.3% in African countries, 3.2–31.5% in central and South American countries, 1.3–13.1% in Asia countries, 0.1–14.1% in Europe, and 0.3–4.3% in North America [4]. In developing countries, 8–19% of diarrheal diseases are attributed to *Cryptosporidium*, and cryptosporidiosis is reported to account for 20% of all cases of diarrhea in children [3,5]. In developed countries, *Cryptosporidium* is less common and accounts for about 9% of diarrheal episodes in children [1].

*Cryptosporidium* oocysts excreted by infected hosts are immediately infectious to other hosts [1]. They can survive for many months in temperate and moist conditions, and are resistant to many common disinfectants, particularly chlorine-based disinfectants [3]. Humans can acquire *Cryptosporidium* infections through the fecal-oral route, either directly or indirectly. Person-to-person transmission is primarily found among children and staff members in nurseries, day-care centers, and schools [6]. In addition, HIV-positive men who have sex with men are reported to have a higher prevalence than HIV-positive drug users (33.3% versus 10.6%) [7]. There have also been about 20 outbreaks of cryptosporidiosis reported in health care facilities [8]. Nosocomial infection by direct and indirect person-to-person transmission is well documented, causing secondary cases among roommates [9] and family members [10]. Animal-to-person transmission primarily occurs among veterinarians and veterinary students as well as other people exposed to agricultural animals and children visiting farms [6]. To date, more than 20 outbreaks related to contact with animals have been reported [6,11–14]. Cattle, especially pre-weaned calves, are considered to be an important source of zoonotic cryptosporidiosis [15]. Humans can also be infected by ingesting water and food contaminated with *Cryptosporidium* oocysts from human and animal feces [1]. To the best of our knowledge, by the end of 2016, at least 524 waterborne outbreaks of cryptosporidiosis had been reported globally, including drinking and recreational water [16–18]. By the end of 2015, at least 26 outbreaks of cryptosporidiosis had been reported to be related to a wide variety of foods, including fruit, vegetables and milk [19]. Therefore, cryptosporidiosis is a globally public health issue, especially waterborne outbreaks.

Extensive genetic variation has been confirmed within the genus *Cryptosporidium*. To date, 38 *Cryptosporidium* species have been accepted and over 40 genotypes are now recognized [20]. More than 20 *Cryptosporidium* species/genotypes have been found in humans [20]. *C*. *hominis* and *C*. *parvum* are the two most common species, being responsible for > 90% of human cases of cryptosporidiosis worldwide [6].

In China, since the first report of human cases of cryptosporidiosis in 1987 [21], *Cryptosporidium* has been attracting increased attention. To date, nearly 200 published papers have documented the occurrence of human *Cryptosporidium* infection/cryptosporidiosis in 29 provinces, autonomous regions, and municipalities. However, there is a lack of a comprehensive understanding of epidemiological status and characteristics of *Cryptosporidium* in humans in China. Therefore, a systematic review was conducted through mining epidemiological data of human *Cryptosporidium* infection/cryptosporidiosis during the past three decades. We presented and analyzed differences in the prevalence of *Cryptosporidium* by province, year, population, living environment and season and possible transmission routes and risk factors as well as genetic characteristics of *Cryptosporidium* in humans. This analysis will be helpful to develop efficient control strategies to intervene with and prevent occurrence of human *Cryptosporidium* infection/cryptosporidiosis in China as well as have a reference effect to other countries.

## Methods

A systematic search was conducted to screen relevant papers published before October 2018, using keywords (*Cryptosporidium* or cryptosporidiosis and China) in PubMed databases via the link (https://www.ncbi.nlm.nih.gov) and using keywords (*Cryptosporidium* or cryptosporidiosis) in three Chinese databases (China National Knowledge Infrastructure, Wanfang, and VIP) via the links (http://www.cnki.net/, http://www.wanfangdata.com.cn/index.html and http://qikan.cqvip.com/). All titles, abstracts and full texts from each of the searches were examined and reviewed to determine whether the studies met inclusion criteria, which reported human *Cryptosporidium* infections/cryptosporidiosis. We excluded duplicated and irrelevant papers including those on animal study, review and pathogenesis as well as case reports of human *Cryptosporidium* infection/cryptosporidiosis. To the goal of assessing the broad epidemiological status and characteristics of *Cryptosporidium* in humans in China, we included papers that met the basic standard requirements and did not exclude some papers based on risk of bias. Our search strategy was illustrated in detail in the subsequent Supporting Information (S1 Flow Diagram).

## Results

Based on the search strategy above, in the end, 164 papers met our inclusion criteria and were eligible for this systematic review, including 14 and 150 papers searched in PubMed and Chinese databases, respectively.

### Prevelance of *Cryptosporidium*

**Fig 1:**
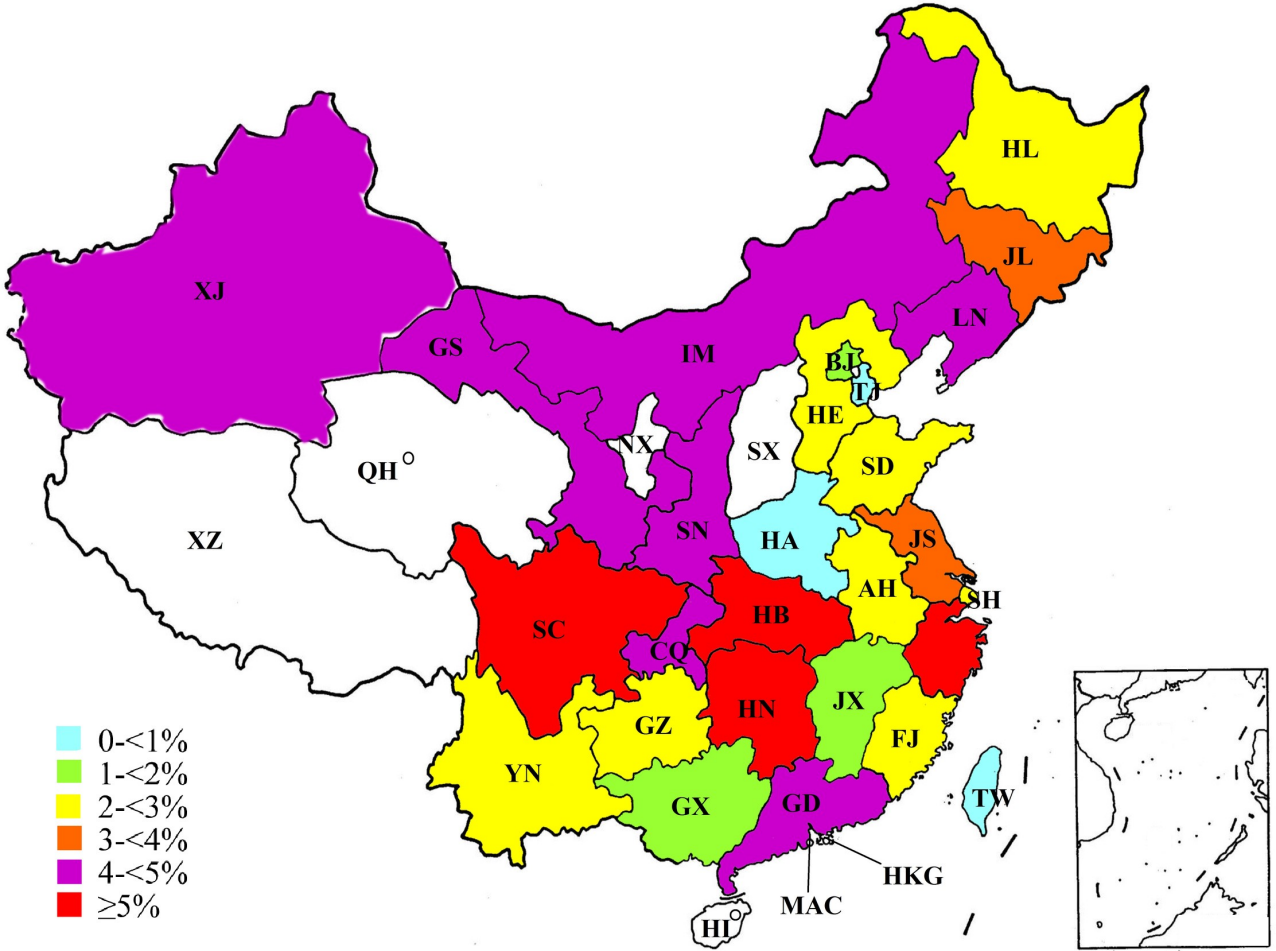
Geographical distribution of human cases of *Cryptosporidium* infection/cryptosporidiosis in China. Variable prevalences are differentiated by color: < 1% in light blue, including Tianjin (TJ), Taiwan (TW), and Henan (HA); 1–< 2% in green, including Beijing (BJ), Guangxi (GX), and Jiangxi (JX); 2–< 3% in yellow, including Anhui (AH), Fujian (FJ), Guizhou (GZ), Hebei (HE), Heilongjiang (HL), Shandong (SD), Shanghai (SH), and Yunnan (YN); 3–< 4% in orange, including Jilin (JL) and Jiangsu (JS); 4–< 5% in purple, including Chongqing (CQ), Guangdong (GD), Gansu (GS), Inner Mongolia (IM), Liaoning (LN), Shaanxi (SN), and Xinjiang (XJ); ≥ 5% in red, including Hubei (HB), Hunan (HN), Sichuan (SC), and Zhejiang (ZJ). Two open circles indicate only one or two cases in the two provinces Hainan (HI) and Qinghai (QH), respectively. No cases were reported in Tibet (XZ), Ningxia (NX), and Shanxi (SX), as well as in two special administrative regions: Hong Kong (HKG) and Macao (MAC). The map was produced from the data presented in S1 Table by using the Microsoft Paint graphics program on our computer, describing vividly an average prevalence for each province.

Since the first case of human cryptosporidiosis was reported in 1987 [21], to date, human cases have been found in 22 provinces, three autonomous regions, and four municipalities, covering the vast majority of Chinese territory (Fig 1 and S1 Table).

**Fig 2:**
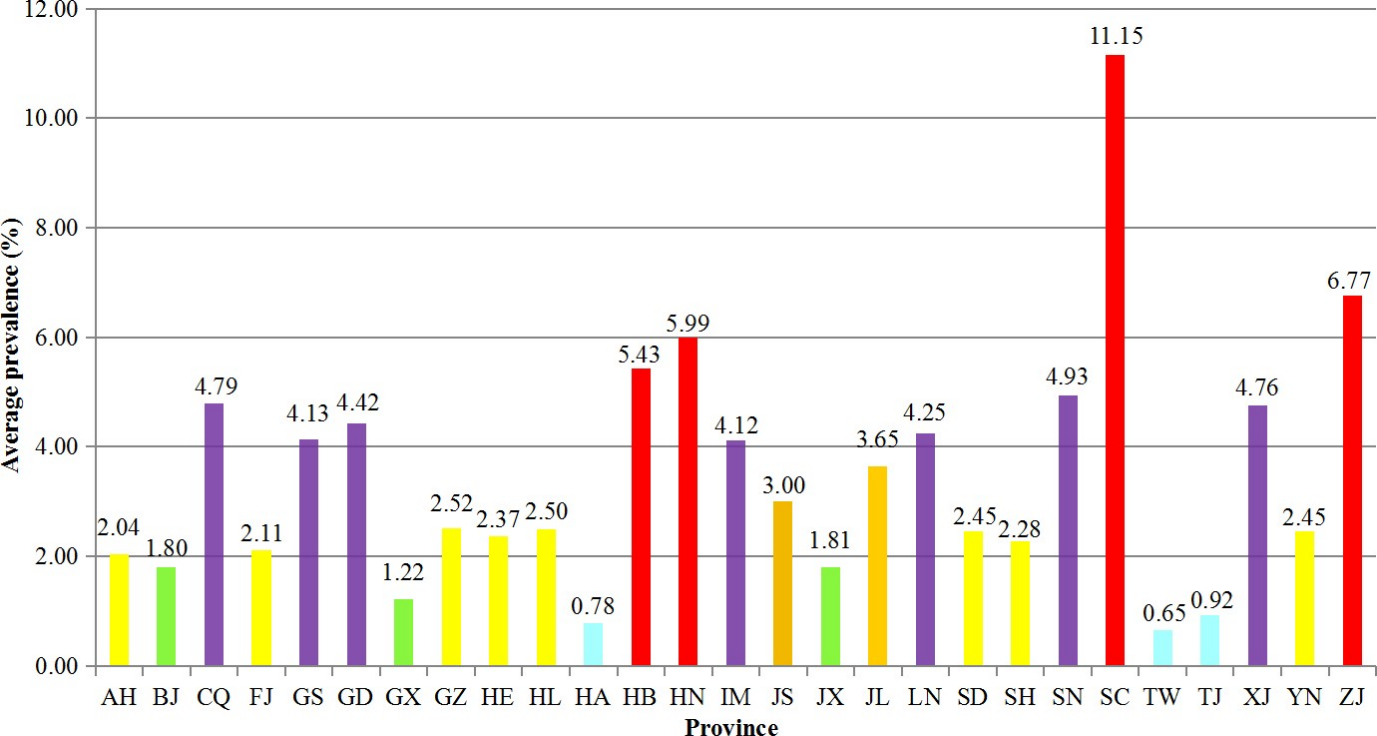
Relative distribution of *Cryptosporidium* in humans across various provinces in China. The bar chart was produced from the data presented in S1 Table, with the colors of strip-shaped bar are consistent with those depicted in Fig 1.

**Fig 3:**
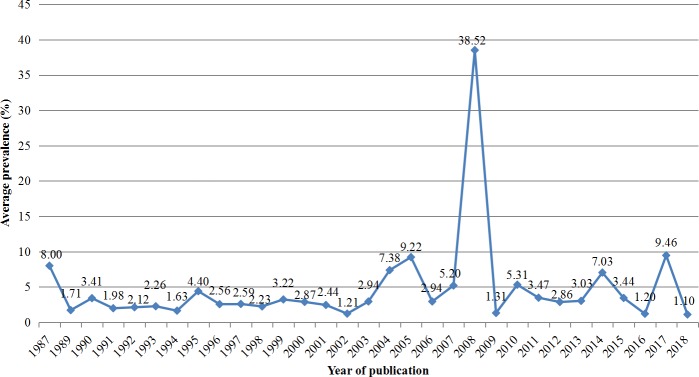
Change tendency over time in the prevalence of *Cryptosporidium* in humans in China during the past three decades. The line chart was produced from the data presented in S2 Table.

Epidemiological investigations of *Cryptosporidium* have been carried out in humans in 27 provincial administrative regions. A total of at least 200,054 people have been involved in investigational studies of *Cryptosporidium* and 5,933 people (2.97%) were diagnosed as having a *Cryptosporidium* infection/cryptosporidiosis. The prevalence of *Cryptosporidium* ranged from 0.65% to 11.15% by province (Fig 2 and S1 Table) and changed slightly in the past three decades (from 1.10% to 9.46%) except 38.52% in 2008 (Fig 3 and S2 Table).

**Fig 4:**
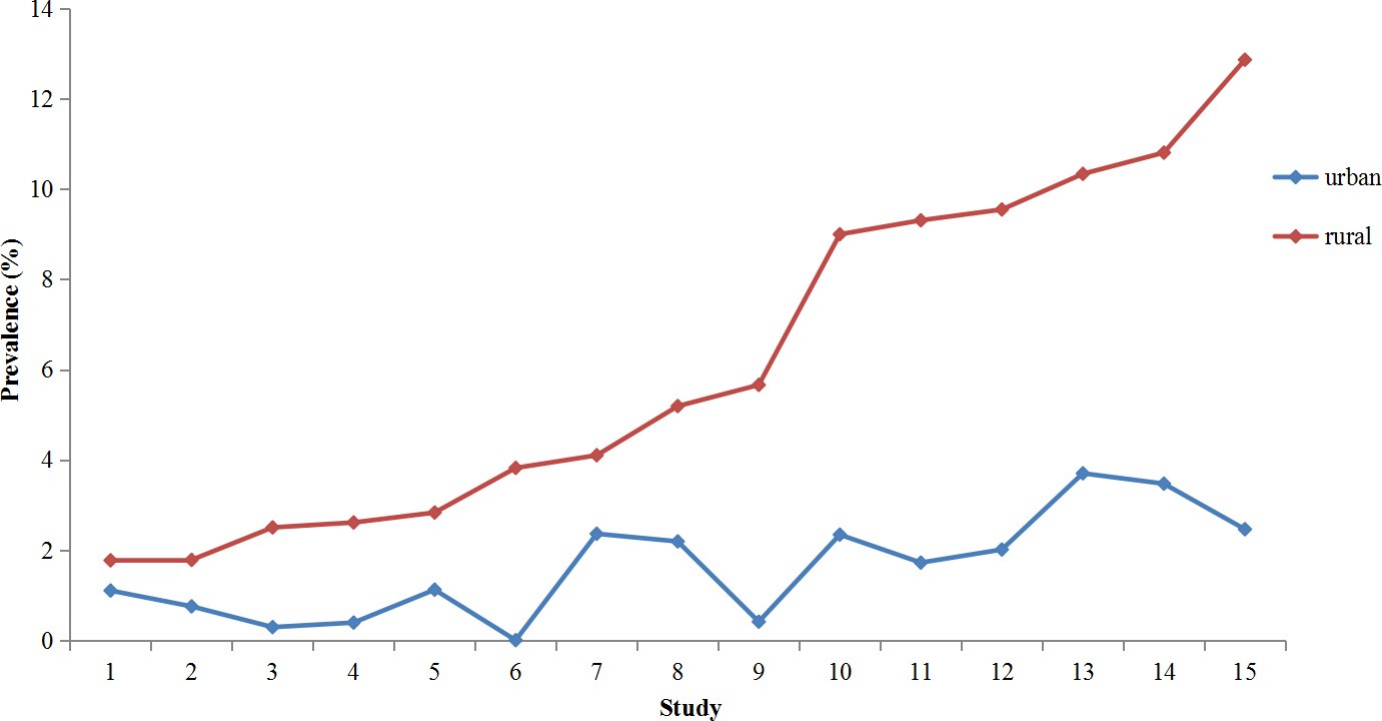
Variations in the prevalence of *Cryptosporidium* in humans by living environment (rural/urban areas) in China. The numbers 1–15 (abscissa) represent 15 studies on comparision of prevalences of *Cryptosporidium* between rural areas and urban areas. The line chart was produced from the data presented in S4 Table.

**Fig 5:**
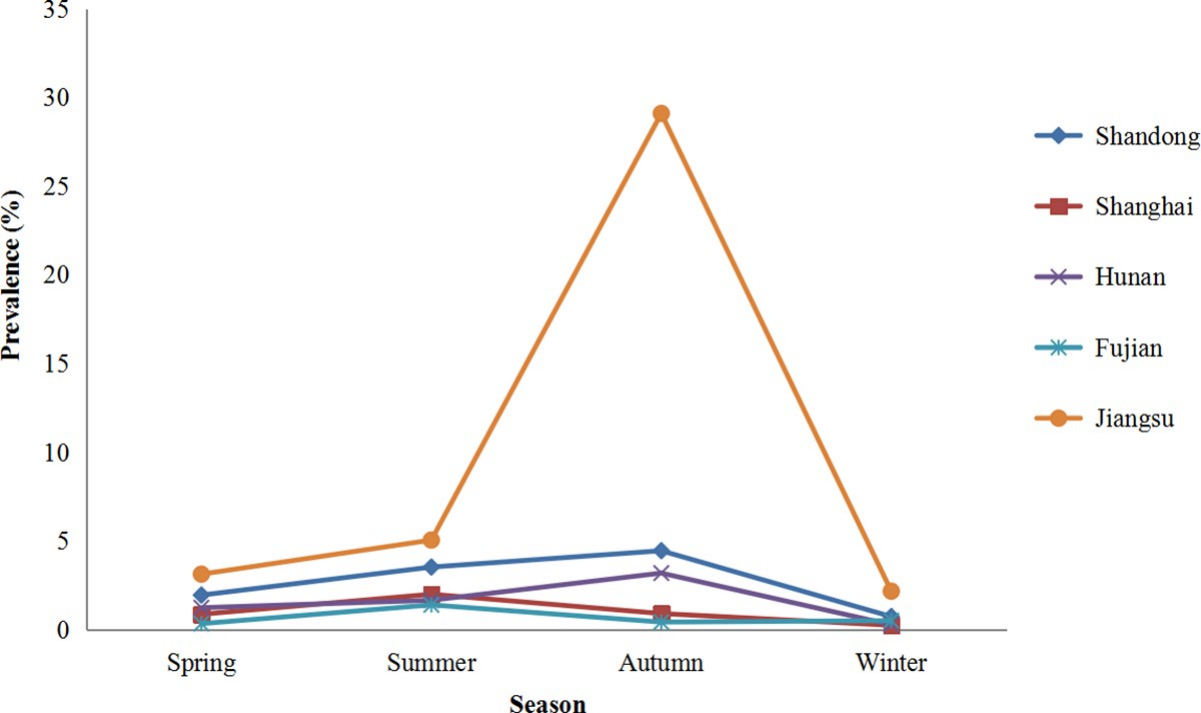
Seasonal variations in the prevalence of *Cryptosporidium* in humans in China. The line chart was produced from the data presented in S5 Table. In China, spring begins in March, summer in June, autumn in September, and winter in December.

Variable prevalences were observed in a few different populations, such as 2.56% (0.57–26.92%) in children under five years (2.04% with diarrhea); 3.68% (0–14.41%) in teenagers; 1.89% (0–29.43%) in adults; 2.75% (0.32–13.49%) in diarrheal patients; 6.57% (0.65–60.0%) in HIV-positive patients (13.40% with diarrhea); 2.28% (0–11.1%) in HIV-negative patients; 4.89% (3.42–21.57%) in HBV–positive patients; 47.79% (10.0–66.67%) in cancer patients; 24.14% (8.57–69.90%) in drug users (S3 Table). People living in rural areas had a significantly higher prevalence of *Cryptosporidium* (1.77–12.87%) than those living in urban areas (0–3.70%) (Fig 4 and S4 Table). Significant seasonal variations were observed in the prevalence of *Cryptosporidium* (*P* < 0.05) based on the five studies, with the peak occurring in summer or autumn (Fig 5 and S5 Table).

### Possible transmission routes and risk factors

In China, there have been only a small number of studies discussing transmission routes and risk factors of *Cryptosporidium* infection/cryptosporidiosis. Some studies indicated the people with contact with animals had a significanyly higher prevalence than others [S1 Table reference list–41,73,92,134,164]. *Cryptosporidium* infection was significantly associated with use of the drinking water supply (6/239 for well water versus 4/861 for tap water and hand pump water) [S1 Table reference list–73]. Drinking unboiled water and eating raw foods were observed to increase *Cryptosporidium* infection of people [S1 Table reference list–70,134,136]. *Cryptosporidium* infections also occurred among family membersand children in nurseries [S1 Table reference list–23,49,53,102,107,122,124,126,152]. There was one outbreak of cryptosporidiosis documented, which occurred in one ward of a pediatric hospital in Shanghai in 2012 [8].

### Genetic characteristics of *Cryptosporidium*

To date, a total of 263 human-derived *Cryptosporidium* isolates were obtained, including 38 from one outbreak. Seven *Cryptosporidium* species were identified, including *C*. *hominis* (127/263, 48.29%), *C*. *andersoni* (59/263, 22.43%), *C*. *parvum* (44/263, 16.73%), *C*. *meleagridis* (22/263, 8.37%), *C*. *felis* (8/263, 3.04%), *C*. *canis* (2/263, 0.76%) and *C*. *suis* (1/263, 0.38%). Some of *C*. *hominis*, *C*. *parvum*, *C*. *meleagridis* isolates were subtyped based on sequence analysis of the gp60 gene. *C*. *hominis* was composed of five subtype families, including Ia (IaA14R4, IaA9R3, and IaA18R4Ia), Ib (IbA16G2, IbA19G2, IbA20G2, IbA22G2), Id (IdA14, IdA19, and IdA21), Ie (IeA12G3T3, IeA13G3T3) and Ig (IgA14). Two subtype families were identified either for *C*. *parvum* or *C*. *meleagridis*, which were composed of IIa and IId (IIdA19G1), and IIIb (IIIbA26G1R1, IIIbA27G1R1, IIIbA29G1R1) and IIIe (IIIeA26G2R1), respectively. Distributions of *Cryptosporidium* species and subtypes in different populations were summarized in Table 1 [8,22–35].

**Table 1:** Distribution of *Cryptosporidium* species and subtypes in different populations in China.

Population	Case number	Species (n)	Subtypes (n)	Ref
Patients with diarrhea	12	*C*. *hominis* (8), *C*. *parvum* (4)	Ia (6), Ib (2), IIa (3), IId (1)	[22]
	2	*C*. *hominis* (2)	IdA21 (2)	[23]
	2	*C*. *parvum* (2)		[24]
	9	*C*. *parvum* (7), *C*. *parvum*+*C*. *homini*s (2)		[25]
	10^a^	*C*. *meleagridis* (10)		[26]
	23	*C*. *andersoni* (21), *C*. *hominis* (2)		[27]
	10	*C*. *parvum* (6), *C*. *felis* (4)		[28]
	34	*C*. *andersoni* (34)		[29]
HBV-positive patients	14	*C*. *parvum* (14)		[24]
HIV-positive patients	6	*C*. *hominis* (2), *C*. *andersoni* (4)		[30]
	10	*C*. *meleagridis* (5), *C*. *hominis* (2), *C*. *parvum* (2), *C*. *suis* (1)	IIIbA26G1R1 (1), IIIbA27G1R1 (1), IIIbA29G1R1 (1), IIIeA26G2R1 (1), Unknown (1), IbA19G2 (2), IIdA19G1 (2)	[31]
	7	*C*. *parvum* (7)		[32]
	4	*C*. *hominis* (2), *C*. *felis* (1), *C*. *meleagridis* (1)		[33]
HIV-negative patients	1	*C*. *hominis* (1)	IeA12G3T3 (1)	[31]
Patients with non-gastrointestinal illnesses	102^b^	*C*. *hominis* (92), *C*. *meleagridis* (6), *C*. *felis* (2), *C*. *canis* (2)	IaA14R4 (36), IdA19 (37), IbA19G2 (1), IdA14 (1), IaA18R4 (1), IgA14 (1)	[8]
Hospitalized patients	10	*C*. *hominis* (9), *C*. *felis* (1)	IbA16G2 (1), IbA19G2 (2), IbA20G2 (3), IaA9R3 (1), IdA21 (2)	[34]
	5	*C*. *hominis* (5)	IbA22G2 (1), IdA14 (1), IeA13G3T3 (1)	[35]
Total	261	*C*. *hominis* (127), *C*. *andersoni* (59), *C*. *parvum* (44), *C*. *meleagridis* (22), *C*. *felis* (8), *C*. *canis* (2), *C*. *suis* (1)	IaA14R4 (36), IaA9R3 (1), IaA18R4 (1), Ia (6), IbA16G2 (1), IbA19G2 (5), IbA20G2 (3), IbA22G2 (1), Ib (2), IdA14 (2), IdA19 (37), IdA21 (4), IeA12G3T3 (1), IeA13G3T3 (1), IgA14 (1), IIa (3), IIdA19G1 (2), IId (1), IIIbA26G1R1 (1), IIIbA27G1R1 (1), IIIbA29G1R1 (1), IIIeA26G2R1 (1), Unknown (1)	

^a^ Children with diarrhea.

^b^ 102 cases include 38 cases from one outbreak occurring in a ward of a pediatric hospital.

## Discussion

*Cryptosporidium* has a wide geographical distribution in China. To date, *Cryptosporidium* has been documented in humans from 29 provinces, autonomous regions, and municipalities, with the prevalence of *Cryptosporidium* ranging from 0.65% to 11.15%. In the past three decades, the prevalence changed slightly (from 1.10% to 9.46%) except 38.52% in 2008. This exception may be related to nearly half of specimens (42.18%, 588/1394) from drug users (S3 Table). Early in 1994, heroin, as one of drugs commonly used by drug users, was noted to cause abnormalities in both humoral and cellular immunity [36]. In China, drug users were observed to have the second highest prevalence (24.14%) of *Cryptosporidium*, only falling behind cancer patients (47.79%) (S3 Table). Cancer patients often experience transient or constant impairments in immunity due to this disease treatment. Currently, chemotherapy is one of the most effective means. Chemotherapy drugs, which are known to be cytotoxic, are reported to possibly down-regulate patients’ immunity and increase the risk of parasitic infections [37]. In Jordan, microscopy results using modified acid fast staining identified a significantly (*P* ≤0.05) higher prevalence of *Cryptosporidium* in pediatric oncology patients with diarrhea (14.4%, 23/160), compared to non-oncology pediatric patients with diarrhea only (5.1%, 7/137).[38]. However, different prevalences of *Cryptosporidium* have been reported in cancer patients in other countries/areas, such as 3.8% in children with cancer in Iran [39] and 13.3% in cancer patients in Brazil [40].

In New South Wales, no *Cryptosporidium* oocysts were detected in 149 stool samples from 60 symptomatic pediatric oncology patients [41]. The difference in prevalence may be related to the extent of the influence of chemotherapy drugs on immune status of cancer patients. Cryptosporidiosis is one of common opportunistic infections among immune-compromised individuals. In some African countries, the prevalence of *Cryptosporidium* in HIV-infected people was reported to be higher than 70.0%, such as 73.6% in Uganda, 79.0% in Nigeria, and 75.6% in South Africa [42]. The introduction of the highly active antiretroviral therapy (HAART) has had a remarkable impact on many opportunistic parasites including *Cryptosporidium*, resulting in a marked reduction in their occurrence and clinical course at least in developed countries [43]. In a multinational cohort study of HIV-positive individuals from Australia and ten European countries, when comparing the periods 1997–2001 and 1994–1996, there was a significant HAART-induced decrease in progression to cryptosporidiosis (3.1% to 0.2%) [44]. In China, HIV-positive patients had a relatively low prevalence of *Cryptosporidium* (6.55%), which may be related to the National Free Antiretroviral Therapy Program (NFATP) initiated in 2002 by the Chinese government. *Cryptosporidium* is a well-established cause of diarrhea among patients with HIV/AIDS and co-infection of this pathogen can increase the mortality [45]. Thus, it is imperative to develop more effective therapeutic agents and vaccines against *Cryptosporidium*. Currently, only nitazoxanide, which is approved by The US Food and Drug Administration, has a treatment effect on non-HIV patients to a certain extent [3]. In fact, in China, some attempts have been made to treat cryptosporidiosis in patients with gastrointestinal illnesses (mostly diarrhea), mainly using allicin (Chinese herbal medicine) or allicin combined with some antibiotics. Therapeutic drugs, dosages and negative conversion ratios of *Cryptosporidium* oocysts in fecal specimens were summarized in S6 Table. Although they showed a better therapeutic effect, it is not clear whether this is a self-healing process or a drug effect, for the vast majority of studies lacked a control group.

Cryptosporidiosis occurs more frequently in infants and children (especially under five years) than in adults both in developed and developing countries [46–48]. This is likely to reflect both exposure and immunity. In developing countries, prevalences of *Cryptosporidium* in diarrheal children under five years were 27% by ELISA in India [49], 28% by microscopy in Ghana [50], 30% by PCR in Tanzania [51], 25% by PCR in Uganda [52], 32% by microscopy in Guatemala [53]. In China, children under five years (2.56%, 269/10491) have a higher prevalence than adults (1.89%, 402/21316) in China (*P* < 0.01). Actually, many factors can influence *Cryptosporidium* prevalences of *Cryptosporidium* in humans. Besides the immune status of the infected hosts, prevalences are also closely related to the detection methods employed. In China, auramine phenol staining and modified acid-fast staining methods are commonly used to detect *Cryptosporidium* oocysts in fecal specimens. However, these traditional staining techniques have been reported to be less specific and sensitive to *Cryptosporidium* oocysts in fecal specimens than immunoassays or PCR-based assays [54]. When a few oocysts are present in human feces and experimental technicians are inexperienced, the prevalence of *Cryptosporidium* is often underestimated. Meanwhile, it is difficult to discriminate some spherical objects similar to *Cryptosporidium* oocysts in size, for yeasts and fecal debris can be stained, especially acid-fast staining method. Thus, the prevalence might also be overestimated.

Infectious diseases including cryptosporidiosis usually disproportionately affect poor populations. In China, most of rural areas are underdeveloped and backward economically. Genarally, people living in rural areas had a significantly higher prevalence of *Cryptosporidium* (1.77–12.87%) than those living in urban areas (0–3.70%). Similar results have also been reported in previous studies from other countries. In countries of the Arab world, higher prevalences of *Cryptosporidium* were found in children living in rural and semi-urban areas than in those residing in urban areas during the decade from 2002 to 2011 [55]. In a retrospective analysis of human cryptosporidiosis cases conducted in New Zealand from 2004 to 2011, the prevalence of cryptosporidiosis in rural areas (yearly average of 71.5 cases per 100,000; 95% confidence interval (CI) 64.6–78.3) was observed to be more than twice that of urban areas (yearly average of 29.2 cases per 100,000; 95% CI 26.9–31.5) [56]. Differences in prevalence might be related to poor sanitation conditions in most of rural areas, a lack of necessary general health knowledge, and health habits of people.

In China, many epidemiological studies discussed the changes in the distribution of human cases of *Cryptosporidium* according to seasons. However, only five studies showed significant seasonal variations in prevalence (*P* < 0.05), with the peak occurring in summer or autumn (Fig 5). In a recent meta-analysis on the seasonality of cryptosporidiosis based on 61 published epidemiological studies, increases in temperature and precipitation were considered to be associated with an increase in the incidence of cryptosporidiosis [57]. It was also observed that in moist tropical climates, precipitation was a strong seasonal driver for cryptosporidiosis; in temperate climates, the incidence of cryptosporidiosis peaked with the increase in temperature [57]. However, the seasonal patterns also vary with geographical locations. In India, the incidence of cryptosporidiosis among children residing in the more temperate northern part of India correlated positively with temperature and negatively with humidity, but correlations were not observed for children residing in the more tropical southern region [58]. Seasonal patterns may vary for different *Cryptosporidium* species. It has been observed that *C*. *hominis* is highly prevalent in autumn in the UK and New Zealand, whereas *C*. *parvum* is more prevalent during spring in Canada, Ireland, and the Netherlands [59]. This is believed to be related to increased exposure to animal oocysts following the calving and lambing season for *C*. *parvum*, and to increased travel, exposure to water, and attendance at day care centers for *C*. *hominis*. China’s climate is varied and complicated because of its vast territory. Meanwhile, there are only a few molecular epidemiological data of *Cryptosporidium* in humans. Thus, the epidemiological characteristics related to the seasonality of *Cryptosporidium* species in China are not sufficiently clear.

*Cryptosporidium* has been detected in at least 240 animal species and the role that animals play in transmission of zoonotic cryptosporidium species to humans has been assessed. Fram animals, especially pre-weaned calves, are considered to be one of main animal reservoir hosts of *Cryptosporidium* in the transmission of human cryptosporidiosis. Contact with infected calves has been implicated as the cause of many small cryptosporidiosis outbreaks in veterinary students, research technicians, and children attending agricultural camps and fairs [13]. In five outbreaks (32 cases) in the UK [60] and one outbreak (40 cases) in Norway [12], all the human cases of cryptosporidiosis were confirmed molecularly to be linked to contact with lambs/goats. However, the zoonotic potential of cats and dogs as common companion animals is considered to be low based on limited or no evidence of *C*. *canis* and *C*. *felis* transmission among people and dogs/cats [61]. In China, the people having close contact with animals were found to have a significanyly higher prevalence than others [S1 Table reference list–41,73,92,134,164], suggestiong the potential of zoonotic transmission.

*Cryptosporidium* oocysts have been detected in various water bodies, which have caused over 524 waterborne outbreaks of cryptosporidiosis worldwide, and most of them are linked to drinking water and recreational water (particularly swimming pools) [16–18]. In China, although no waterborne outbreaks have been reported, *Cryptosporidium* oocysts have been detected in source and tap water [62–64] as well as swimming pools [65,66]. In a study conducted in Shanghai, well water as the drinking water supply had higher risk for human *Cryptosporidium* infection than tap water and hand pump water [S1 Table reference list–73]. In addition, drinking unboiled water was considred to be another risk factor [S1 Table reference list–70]. Based on the public health significance of *Cryptosporidium*, this pathogen has been listed as one of microbial contaminant indicators in Chinese Standards for Drinking Water Quality early in 2006 (GB5749-2006). Although waterborne cryptosporidiosis is recognized as a major problem for the drinking water industry after large outbreaks in the 1980s and 1990s, more recently, recreational water, particularly treated water venues such as swimming pools, has emerged as the primary waterborne route of transmission [16–18]. The facts indicate a severe challenge to improvement of the safety of swimming pool water. The oocysts have also been identified as contaminations in different types of food, mainly on numerous fresh vegetables and fruits, and at least 26 foodborne outbreaks have been reported worldwide [19]. These foods are often eaten raw or after minimal thermal treatment, increasing the possibility of transmission of cryptosporidiosis. In China, there were no reports of foods contaminated with *Crysporidium* oocysts. However, two studies indicated that eating raw foods increased *Cryptosporidium* infection of people [S1 Table reference list–134,136].

In China, *Cryptosporidium* infections were also observed among family members, and children in nurseries, indicating the possibility of person-to-person transmission [S1 Table reference list–23,49,53,102,107,122,124,126,152]. Remarkably, there was one outbreak of cryptosporidiosis occurring in one ward of a pediatric hospital in Shanghai in 2012 [8]. This outbreak lasted more than one year and affected 51.4% (38/74) of infant patients, and *C*. *hominis* was identified. However, the source of this outbreak is unclear. Most of the patients were examined for *Cryptosporidium* only once, and many of the specimens were not submitted immediately after patients were hospitalized. The likelihood of widespread foodborne and waterborne transmission of cryptosporidiosis was small because the children in this ward and other wards shared the same source of food and drinking water. Meanwhile, the likelihood of direct transmission of cryptosporidiosis was also small because 80% of patients were less than one year old and mostly stayed in cribs and beds. Therefore, the authors speculated that poor diaper changing and hand washing practices by caregivers could have been responsible for the persistence of *C*. *hominis* infections. Although the source of the infection was not determined, this hospital took measures to reduce hospital-acquired infections, including better training of caregivers and moving the ward where the outbreak occurred to a new location. The possibility that cryptosporidiosis may be introduced into and transmitted within hospitals requires particular attention in the future.

Molecular epidemiological data of *Cryptosporidium* have revealed that the distribution of *Cryptosporidium* species in humans is different among geographic areas. Since genotyping and subtyping tools have been used in identification of human cases of cryptosporidiosis in 2011 [35], to date, genetic characteristics of 263 human-derived *Cryptosporidium* isolates were obtained, and seven *Cryptosporidium* species have been identified, including *C*. *hominis*, *C*. *parvum*, *C*. *andersoni*, *C*. *meleagridis*, *C*. *felis*, *C*. *canis*, and *C*. *suis* (Table 1).

*C*. *hominis* and *C*. *parvu*m are the two most common species in humans and are responsible for greater than 90% of human cases of cryptosporidiosis in most countries [6]. Current molecular epidemiological data have demonstrated that the distribution of the two major *Cryptosporidium* species in humans actually differs geographically. In European countries and New Zealand, both species are frequently detected in humans. In the Middle East, *C*. *parvum* is also the dominant species. In contrast, *C*. *hominis* is responsible for more infections than *C*. *parvum* in other industrialized nations and in developing countries [3,20]. In China, 48.29% (127/263) and 16.73% (44/263) of human cases are caused by *C*. *hominis* and for *C*. *parvum*, respectively. At the subtype level, based on sequence analysis of the gp60 gene, five subtype families (Ia, Ib, Id, Ie, and Ig) have been identified for *C*. *hominis* in China, composed of IaA14R4, IaA9R3, and IaA18R4; IbA16G2, IbA19G2, IbA20G2, IbA22G2; IdA14, IdA19, and IdA21 (Table 1). These subtypes are rarely detected in humans in other countries [67] and some of them, such as IbA16G2, IbA19G2, and IbA20G2, are restricted to China [8,34]. They are different from the two common Ib subtypes (IbA9G3 and IbA10G2) in humans, with the former being distributed worldwide and the latter being mostly observed in certain developing countries [20]. Compared with *C*. *hominis*, there is less information about *C*. *parvum* subtypes in humans in China. Only two subtype families (IIa and IId) were identified and only IIdA19G1 has been found (Table 1). *C*. *parvum* is the most important zoonotic *Cryptosporidium* species, with a broad host range, and can be found in young farmed animals. Calves, especially pre-weaned calves, are the main animal reservoir hosts of this species and pose the most significant threat to environmental contamination and transmission to humans. In China, in addition to cattle, *C*. *parvum* has been found in golden takins, sheep, goats, yaks, horses, and donkeys, suggesting the possibility of zoonotic transmission [67].

Surprisingly, *C*. *andersoni* was identified at a high frequency (22.43%, 59/263) in humans in China. This species was identified diarrheal outpatients from Jiangsu (n = 21) and Shanghai (n = 34) and in HIV/AIDS patients [27,29,30]. Besides China, this species has also been identified in a few sporadic cases of cryptosporidiosis in the UK [68], Malawi [69], Australia [70], Iran [71], France [72], and India [73], and is a predominant species (79.59%,78/98) in diarrheal patients in India [73]. *C*. *andersoni* is actually the most common *Cryptosporidium* species responsible for cattle cryptosporidiosis in yearlings and adults. Recently, with the establishment of the multilocus sequence typing (MLST) tool used for subtyping *C*. *andersoni* [74], the MLST subtypes and population genetic structure of *C*. *andersoni* from animals have been analyzed, including those from cattle, sheep, horses, golden takins, monkeys, camels, ostriches, and hamsters [75–84]. These MLST data of animal-derived *C*. *andersoni* isolates will be helpful in the future for source attribution of infection/contamination of *C*. *andersoni* and to understand its transmission dynamics in humans.

Some *Cryptosporidium* species associated with various animals have also been identified in humans in China, including *C*. *meleagridis*, *C*. *felis*, *C*. *canis*, and *C*. *suis*. *C*. *meleagridi*s. *C*. *meleagridi*s is recognized as the third most prevalent *Cryptosporidium* species infecting humans after *C*. *hominis* and *C*. *parvum* [85]. However, in some countries/areas, *C*. *meleagridis* is as prevalent in humans as *C*. *parvum*, and is responsible for 10–20% of human cryptosporidiosis cases, particularly in Lima (Peru) and Bangkok (Thailand) [86–88]. In a study conducted in Peru, *C*. *meleagridis* was more commonly identified in humans than *C*. *parvum* [89]. *C*. *meleagridis* has been detected in wide range of avian species, and is occasionally found in some mammal hosts (minks, cattle, wallabies, gorillas, and dogs) as well as some bivalves [90]. Early in 2010, zoonotic transmission of *C*. *meleagridis* was reported in Sweden based on identical *C*. *meleagridis* sequences of the SSU rRNA and 70 kDa Heat Shock Protein (HSP) genes in human and chicken fecal samples [91]. The likely occurrence of cross transmission of *C*. *meleagridis* between birds and humans has been further evidenced by analyzing the genetic diversity and population structure of *C*. *meleagridis* using MLST tool [92]. In China, to date, *C*. *meleagridis* has been detected in various bird species, including chickens [93], pigeons [94], quails [95], and a variety of pet birds [96], as well as in two mammalian species (minks and calves) [90,97]. *C*. *felis* and *C*. *canis* are frequently reported in studies in developing countries, and show a low ratio in human cases of *Cryptosporidium* infection [3]. In China, to date, only eight and two human cases of *Cryptosporidium* infection were attributed to *C*. *felis* and *C*. *canis*, respectively [8,28,33]. The two *Cryptosporidium* species are mainly associated with cats and dogs, respectively [3]. Besides cats and dogs, *C*. *felis* and *C*. *canis* have also been found in other feline and canine animals, such as foxes, raccoon dogs, manuls [67], and minks [90], suggesting a possible risk of zoonotic transmission. *C*. *suis* is rarely detected in humans. To date, *C*. *suis* has only been identified in HIV-positive patients in Peru and China [31,87,98], as well as diarrheal persons in the UK and Madagascar [68,99]. *C*. *suis* is mainly found in pigs. In China, pigs as the major economic animals are reported to be commonly infected with *C*. *suis* [100–102]. Pig cryptosporidiosis should receive more attention, for pig cryptosporidiosis is not only a veterinarian issue, but may be important for public health. *C*. *suis* has been detected in drinking source water [62,64,103,104]. Thus, it is necessary to develop better farm management systems to reduce environmental contamination of zoonotic agents, and prevent the occurrence of zoonotic transmissions of human cryptosporidiosis caused by *C*. *suis*.

## Conclusions

This is the first comprehensively retrospective epidemiological analysis of human *Cryptosporidium* infection/cryptosporidiosis in China since the first report in 1987, reflecting current epidemiological status and characteristics of this parasitic disease. Meanwhile, these data will be helpful to develop efficient control strategies to intervene with and prevent the occurrence of human *Cryptosporidium* infection/cryptosporidiosis in China and have a reference effect to other countries. Further studies should focus on addressing a high frequency of *C*. *andersoni* in humans and a new challenge with respect to cryptosporidiosis with an increasing population of elderly people and patients with immunosuppressive diseases. Based on the Manhattan Principles on “One World, One Health”, it is necessary to require interdisciplinary and cross-sectoral approaches to cryptosporidiosis prevention, surveillance, monitoring, control and mitigation as well as to environmental conservation more broadly.

In this systematic review, there are also some limitations. In 89.02% (146/164) epidemiological studies in China covering 181,807 people, *Cryptosporidium* infection/cryptosporidiosis was confirmed based on microscopy observation after staining. Meanwhile, the analysis results are also affected by the absence of epidemiological data in some provinces. In fact, they may be related to weak surveillance, rather than real absence of *Cryptosporidium* infection/cryptosporidiosis there.

## Supporting information

S1 Flow Diagram (DOC)

S1 Table Prevalence of *Cryptosporidium* in humans by province in China. (DOCX)

S2 Table Prevalence of *Cryptosporidium* in humans by year of publication in China. (DOCX)

S3 Table Prevalence of *Cryptosporidium* by population in China. (DOCX)

S4 Table Prevalence of *Cryptosporidium* in humans by living environment (rural/urban areas) in China. (DOCX)

S5 Table Prevalence of *Cryptosporidium* in humans by season in China. (DOCX)

S6 Table Drugs, dosages and negative conversion ratios of oocysts in fecal specimens under microscopy. (DOCX)

S1 Checklist (DOCX)
