# Supplementary material for: A retrospective epidemiological analysis of human Cryptosporidium infection in China during the past three decades (1987-2018)
Source: PLoS Negl Trop Dis. 2020 Mar 30;14(3):e0008146. doi: 10.1371/journal.pntd.0008146 (PMC7145189; doi:10.1371/journal.pntd.0008146)
Supplement: S1 Flow Diagram — (DOC) [file pntd.0008146.s001.doc]

S1 Flow diagram

**Screening**

**Included**

**Eligibility**

**Identification**

Records identified through PubMed database searching
(n = 205)

Records identified through three Chinese databases searching after duplicates removed
(n = 1468)

Records after duplicates removed
(n = 1642)

Irrelevant records on animal study, review and pathogenesis excluded

(n = 1452)

Papers assessed for eligibility by examining titles, abstracts and full texts

(n = 190)

164 papers included in final analysis
(14 in English and 150 in Chinese)

(n = 203)

Case reports excluded

(n = 26)
