# Supplementary material for: A retrospective epidemiological analysis of human Cryptosporidium infection in China during the past three decades (1987-2018)
Source: PLoS Negl Trop Dis. 2020 Mar 30;14(3):e0008146. doi: 10.1371/journal.pntd.0008146 (PMC7145189; doi:10.1371/journal.pntd.0008146)
Supplement: S1 Table — (DOCX) [file pntd.0008146.s002.docx]

S1 Table. Prevalence of *Cryptosporidium* in humans by province in China.

| **Province (Abbreviation)^a^** | **Examined no** | **Positive no.** | **Prevalence (%)** | **Ref** |
| --- | --- | --- | --- | --- |
| Anhui (AH) | 203 | 7 | 3.45 | [1] |
|  | 827 | 46 | 5.56 | [2] |
|  | 4048 | 54 | 1.33 | [3] |
|  | 5421 | 41 | 0.76 | [4] |
|  | 1204 | 42 | 3.49 | [5] |
|  | 889 | 28 | 3.15 | [6] |
|  | 342 | 32 | 9.36 | [7] |
|  | 635 | 15 | 2.36 | [8] |
|  | 5421 | 74 | 1.37 | [9] |
|  | 720 | 32 | 4.44 | [10] |
|  | 302 | 21 | 6.95 | [11] |
|  | 3498 | 67 | 1.92 | [12] |
|  | 605 | 34 | 5.62 | [13] |
| **Subtotal** | **24115** | **493** | **2.04** **(0.76–9.36)** | **n = 13** |
| Beijing (BJ) | 592 | 5 | 0.84 | [14] |
|  | 2344 | 30 | 1.28 | [15] |
|  | 67 | 4 | 5.97 | [16] |
|  | 125 | 11 | 8.80 | [17] |
|  | 30 | 7 | 23.33 | [18] |
| **Subtotal** | **3158** | **57** | **1.80** **(0.84–23.33)** | **n = 5** |
| Chongqing (CQ) | 250 | 20 | 8.00 | [19] |
|  | 830 | 34 | 4.10 | [20] |
|  | 649 | 46 | 7.09 | [21] |
|  | 1238 | 42 | 3.39 | [22] |
| **Subtotal** | **2967** | **142** | **4.79** **(3.39–8.00)** | **n = 4** |
| Fujian | 161 | 16 | 9.94 | [23] |
|  | 62 | 8 | 12.90 | [24] |
|  | 359 | 6 | 1.67 | [25] |
|  | 385 | 31 | 8.05 | [26] |
|  | 210 | 8 | 3.81 | [27] |
|  | 3116 | 20 | 0.64 | [28] |
|  | 248 | 7 | 2.82 | [29] |
|  | 191 | 4 | 2.09 | [30] |
| **Subtotal** | **4732** | **100** | **2.11 (0.64–12.90)** | **n = 8** |
| Gansu (GS) | 37 | 1 | 2.70 | [31] |
|  | 186 | 13 | 6.99 | [32] |
|  | 1840 | 41 | 2.23 | [33] |
|  | 580 | 54 | 9.31 | [34] |
|  | 600 | 25 | 4.17 | [35] |
| **Subtotal** | **3243** | **134** | **4.13** **(2.23–9.31)** | **n = 5** |
| Guangdong (GD) | 126 | 5 | 3.97 | [36] |
|  | 182 | 5 | 2.75 | [37] |
|  | 271 | 4 | 1.48 | [38] |
|  | 42 | 4 | 9.52 | [39] |
|  | 693 | 16 | 2.31 | [40] |
|  | 556 | 19 | 3.42 | [41] |
|  | 630 | 98 | 15.56 | [42] |
|  | 1837 | 44 | 2.40 | [43] |
|  | 348 | 12 | 3.45 | [44] |
| **Subtotal** | **4685** | **207** | **4.42** **(1.48–15.56)** | **n = 9** |
| Guangxi (GX) | 54 | 1 | 1.85 | [45] |
|  | 73 | 1 | 1.37 | [46] |
|  | 258 | 6 | 2.33 | [47] |
|  | 435 | 2 | 0.46 | [48] |
| **Subtotal** | **820** | **10** | **1.22 (0.46–2.33)** | **n = 4** |
| Guizhou (GZ) | 335 | 23 | 6.86 | [49] |
|  | 1946 | 41 | 2.11 | [50] |
|  | 150 | 1 | 0.67 | [51] |
|  | 1739 | 40 | 2.30 | [52] |
| **Subtotal** | **4170** | **105** | **2.52 (0.67–6.86)** | **n = 4** |
| Hebei (HE) | 4889 | 106 | 2.17 | [53] |
|  | 107 | 2 | 1.87 | [54] |
|  | 49 | 3 | 6.12 | [55] |
|  | 149 | 9 | 6.04 | [56] |
|  | 69 | 2 | 2.90 | [57] |
|  | 130 | 6 | 4.62 | [58] |
| **Subtotal** | **5393** | **128** | **2.37** **(1.87–6.12)** | **n = 6** |
| Heilongjiang (HL) | 931 | 13 | 1.40 | [59] |
|  | 330 | 11 | 3.33 | [60] |
|  | 58 | 9 | 15.52 | [61] |
| **Subtotal** | **1319** | **33** | **2.50** **(1.39–15.5)** | **n = 3** |
| Henan (HA) | 149 | 24 | 16.11 | [62] |
|  | 388 | 10 | 2.58 | [63] |
|  | 483 | 12 | 2.48 | [64] |
|  | 1074 | 12 | 1.12 | [65] |
|  | 699 | 4 | 0.57 | [66] |
|  | 912 | 2 | 0.22 | [67] |
|  | 6093 | 6 | 0.10 | [68] |
|  | 1949 | 3 | 0.15 | [69] |
|  | 4357 | 43 | 0.99 | [70] |
|  | 1996 | 2 | 0.10 | [71] |
|  | 158 | 24 | 15.19 | [72] |
|  | 1366 | 11 | 0.81 | [73] |
| **Subtotal** | **19624** | **153** | **0.78 (0.10–16.11)** | **n = 12** |
| Hubei (HB) | 941 | 62 | 6.59 | [74] |
|  | 1118 | 51 | 4.56 | [75] |
|  | 2549 | 119 | 4.67 | [76] |
|  | 217 | 106 | 48.85 | [77] |
|  | 1128 | 36 | 3.19 | [78] |
|  | 1035 | 58 | 5.60 | [79] |
|  | 1973 | 79 | 4.00 | [43] |
|  | 298 | 9 | 3.02 | [80] |
|  | 500 | 10 | 2.00 | [81] |
| **Subtotal** | **9759** | **530** | **5.43** **(2.00–48.85)** | **n = 9** |
| Hunan (HN) | 3739 | 69 | 1.85 | [82] |
|  | 250 | 20 | 8.00 | [83] |
|  | 102 | 5 | 4.90 | [84] |
|  | 52 | 2 | 3.85 | [85] |
|  | 294 | 27 | 9.18 | [86] |
|  | 903 | 172 | 19.05 | [87] |
|  | 356 | 3 | 0.84 | [88] |
|  | 370 | 34 | 9.19 | [89] |
|  | 903 | 151 | 16.72 | [90] |
|  | 179 | 34 | 18.99 | [91] |
|  | 2134 | 41 | 1.92 | [92] |
|  | 152 | 20 | 13.16 | [93] |
|  | 480 | 16 | 3.33 | [94] |
| **Subtotal** | **9914** | **594** | **5.99 (0.84–19.05)** | **n = 13** |
| Inner Mongolia (IM) | 140 | 5 | 3.57 | [95] |
|  | 1002 | 42 | 4.19 | [96] |
| **Subtotal** | **1142** | **47** | **4.12** **(3.57–4.19)** | **n = 2** |
| Jiangsu (JS) | 1014 | 13 | 1.28 | [97] |
|  | 478 | 20 | 4.18 | [98] |
|  | 4582 | 59 | 1.29 | [99] |
|  | 1648 | 18 | 1.09 | [43] |
|  | 5124 | 163 | 3.18 | [100] |
|  | 232 | 23 | 9.91 | [101] |
|  | 230 | 12 | 5.22 | [102] |
|  | 394 | 14 | 3.55 | [103] |
|  | 1065 | 1 | 0.09 | [104] |
|  | 3488 | 94 | 2.69 | [105] |
|  | 7393 | 136 | 1.84 | [106] |
|  | 6498 | 163 | 2.51 | [107] |
|  | 2018 | 16 | 0.79 | [108] |
|  | 1000 | 13 | 1.30 | [109] |
|  | 314 | 1 | 0.32 | [110] |
|  | 5089 | 89 | 1.75 | [111] |
|  | 480 | 10 | 2.08 | [112] |
|  | 972 | 524 | 53.91 | [113] |
|  | 2268 | 22 | 0.97 | [114] |
|  | 1651 | 18 | 1.09 | [115] |
|  | 1637 | 18 | 1.10 | [116] |
| **Subtotal** | **47575** | **1427** | **3.00** **(0.09–53.91)** | **n = 21** |
| Jiangxi (JX) | 121 | 2 | 1.65 | [117] |
|  | 210 | 4 | 1.90 | [118] |
| **Subtotal** | **331** | **6** | **1.81 (1.65–1.9)** | **n = 2** |
| Jilin (JL) | 91 | 13 | 14.29 | [119] |
|  | 2046 | 65 | 3.18 | [120] |
| **Subtotal** | **2137** | **78** | **3.65** **(3.18–14.29)** | **n = 2** |
| Liaoning (LN) | 353 | 15 | 4.25 | [121] |
| **Subtotal** | **353** | **15** | **4.25** | **n = 1** |
| Shandong (SD) | 1835 | 15 | 0.82 | [122] |
|  | 969 | 26 | 2.68 | [123] |
|  | 2256 | 61 | 2.70 | [124] |
|  | 2854 | 33 | 1.16 | [125] |
|  | 3655 | 87 | 2.38 | [126] |
|  | 3562 | 84 | 2.36 | [127] |
|  | 1943 | 55 | 2.83 | [128] |
|  | 3729 | 75 | 2.01 | [129] |
|  | 237 | 6 | 2.53 | [130] |
|  | 108 | 72 | 66.67 | [131] |
|  | 112 | 7 | 6.25 | [132] |
|  | 23 | 1 | 4.35 | [133] |
| **Subtotal** | **21283** | **522** | **2.45 (0.82–66.67)** | **n = 12** |
| Shanghai (SH) | 500 | 24 | 4.80 | [134] |
|  | 393 | 29 | 7.38 | [135] |
|  | 2817 | 37 | 1.31 | [136] |
|  | 6284 | 102 | 1.62 | [137] |
|  | 109 | 10 | 9.17 | [138] |
|  | 252 | 34 | 13.49 | [139] |
| **Subtotal** | **10355** | **236** | **2.28 (1.31–13.49)** | **n = 6** |
| Shaanxi (SN) | 190 | 3 | 1.58 | [140] |
|  | 200 | 3 | 1.50 | [141] |
|  | 462 | 36 | 7.79 | [142] |
| **Subtotal** | **852** | **42** | **4.93 (1.50–7.79)** | **n = 3** |
| Sichuan (SC) | 406 | 12 | 2.96 | [143] |
|  | 79 | 13 | 16.46 | [144] |
|  | 615 | 74 | 12.03 | [145] |
|  | 1635 | 206 | 12.60 | [146] |
| **Subtotal** | **2735** | **305** | **11.15** **(2.96–16.46)** | **n = 4** |
| Taiwan (TW) | 1376 | 9 | 0.65 | [147] |
| **Subtotal** | **1376** | **9** | **0.65** | **n = 1** |
| Tianjin (TJ) | 1200 | 11 | 0.92 | [148] |
| **Subtotal** | **1200** | **11** | **0.92** | **n = 1** |
| Xinjiang (XJ) | 1124 | 53 | 4.72 | [149] |
|  | 37 | 4 | 10.81 | [62] |
|  | 49 | 4 | 8.16 | [72] |
|  | 114 | 2 | 1.75 | [150] |
| **Subtotal** | **1324** | **63** | **4.76** **(1.75–10.81)** | **n = 4** |
| Yunnan (YN) | 73 | 9 | 12.33 | [151] |
|  | 4493 | 126 | 2.80 | [152] |
|  | 2450 | 33 | 1.35 | [153] |
|  | 87 | 2 | 2.30 | [154] |
|  | 524 | 3 | 0.57 | [155] |
|  | 500 | 84 | 16.80 | [156] |
|  | 30 | 18 | 60.00 | [157] |
|  | 378 | 20 | 5.29 | [158] |
|  | 4405 | 20 | 0.45 | [159] |
|  | 86 | 4 | 4.65 | [36] |
| **Subtotal** | **13026** | **319** | **2.45 (0.45–60.00)** | **n = 10** |
| Zhejiang (ZJ) | 1060 | 60 | 5.66 | [160] |
|  | 548 | 57 | 10.40 | [161] |
|  | 300 | 26 | 8.67 | [162] |
|  | 200 | 9 | 4.50 | [163] |
|  | 358 | 15 | 4.19 | [164] |
| **Subtotal** | **2466** | **167** | **6.77 (4.19–10.40)** | **n = 5** |
| **Total** | **200054** | **5933** | **2.97** | **n = 164^b^** |

^a^ Two reports from Hainan (HI) and Qinghai (QH) were not included in S1 Table based on two and one cases found there, respectively.

^b^ Due to four reports covering epidemiological date from some parts of two or three provinces, each of the four reports is only counted once in the total number.

**References**

1. Lu J, Li CP, Yang ZH, Zhan LH. An epidemiological survey of cryptosporidiosis in Huainan district. J Huainan Inst Technol. 2001;21(4):71–2. Chinese.
2. Cai R, Li CP, Wang J, Xu LF, He Y. An epidemiology survey of cryptosporidiosis with diarrhea in Huainan area. J Trop Dis Parasitol. 2003;1(1):26–8. Chinese.
3. Xu LF, Wang J, Wang KX, Li CP. *Cryptosporidium* infection among students in Anhui Province. Chin Sch Health. 2003;24(4):347–8. Chinese.
4. Wang KX, Xu LF, Cai R, Yang QG, He J. Detection of mIL–2R and T subsets in PBMC of the patients infected by *Cryptosporidium parvum*. Chin J Zoonoses. 2003;19(5):95–7. Chinese.
5. Lu J, Li CP. The Survey of *Cryptosporidium* infection among young children in kindergartens in Anhui Province. Chin J Parasitol Parasit Dis. 2004;22(6):13–5. Chinese.
6. Cui YB, Xing YR. Detection of PA and Fn in serum of children with *Cryptosporidium* oocyst positive in fecal specimens. Chin J Parasitol Parasitic Dis. 2004;4:42. Chinese.
7. Liu DH, Wang TP, Li YF, Yang WP, Guo JD. Co–infection status of HIV and *Cryptosporidium* in Xuancheng City. Parasitoses Infect Dis. 2013;11(4):197–200. Chinese.
8. Wang QQ, Guo JD, Cao ZG, Wang QZ, Liu DH, Wang TP. Investigation on human *Cryptosporidium* infection in local area of Anhui. Chin J Schisto Control. 2015;27(3):263–7. Chinese.
9. Wang KX, Li CP, Wang J, Pan BR. Epidemiological survey of cryptosporidiosis in Anhui Province China. World J Gastroenterol. 2002;8(2):371–4.
10. Tian LG, Wang TP, Cheng GJ, Wang FF, Tong XM, Guo J, et al. Cross–section study on co–infection of HIV and *Cryptosporidium*. Chin J Schisto Control. 2012;24(1):54–7. Chinese.
11. Tian LG, Wang TP, Cheng GJ, Wang FF, Tong XM, Guo J, et al. Cross–section study on co–infection of HIV and *Cryptosporidium*. Chin J Schisto Control. 2012;24(1):54–7. Chinese.
12. Zhu F. Cryptosporidiosis in children in Wuhu. Chin J Epidemiol. 1991;12(5):286–8. Chinese.
13. Tian LG, Chen JX, Wang TP, Cheng GJ, Steinmann P, Wang FF, et al. Co–infection of HIV and intestinal parasites in rural area of China. Parasit Vectors. 2012;13(5):36.
14. Feng W, Gu X, Sui W, Zhang M, Lu B, Wang M, et al. The application and epidemiological research of xTAG GPP multiplex PCR in the diagnosis of infectious diarrhea. Natl Med J China. 2015 Feb 10;95(6):435–9. Chinese.
15. Wang HF, Liu J, Yu JF, Sun F. A Rapid Immunoassay for detection of *Cryptosporidium parvum* and *Giardia lamblia* antigens in human stool and its application. J Mod Lab Med. 2010;25(4):65–6. Chinese.
16. Li M, Liu Y, Wang HZ, Li J, Jiao BX. *Cryptosporidium* infection among HIV/AIDS patients with chronic diarrhea in Beijing. Chin J Public Health. 2012,28(8):1099–101. Chinese.
17. Wang HZ, Guo JJ, Li M, Li RH, Zhang Y, Lu Y, et al. Study of Cryptosporidium infection in HIV/AIDS patients with chronic diarrhea. Int J Lab Med. 2015;36(22):3300–3301. Chinese.
18. Lu S, Wang F, Zhang K, Xu L. Study on genetic approach in the detection of *Cryptosporidium parvum* and *Giardia lamblia* in acquired immunodeficiency syndrom patients. Chin J Epidemiol. 2006;27(10):884–8. Chinese.
19. Zhou YH. *Cryptosporidium* infection and enteritis in humans in Chongqing. J Chongqing Med Univ. 1987;1:3. Chinese.
20. Li WM, Cui FW, Zheng CZ, Yang Y. Clinical study on crptosporidial enteritis and its treatment with Chinese herb medicines in children. Acta Acad Med Mil Tertiae. 1993;15(6):513–6. Chinese.
21. Zheng CZ, Li WM, Cui FW, Cui SX, Yang Y. Laboratory study on diagnosis of cryptosporidiosis in children with diarrhea. Chin J Pediatr. 2000;6:32–5. Chinese.
22. Zhang RP, Li XY, Li H, Pu CW, Feng L, Feng CY. *Cryptosporidium* in eastern Chongqing. Parasitoses Infect Dis. 2012;10(2):72–4. Chinese.
23. Su QP, Chen DG, Hua XL, Chen S, Zhao ZQ, Huang MH, et al. Cryptosporidiosis from infants in Fuzhou region. Chin J Zoonoses. 1989;5:35–6. Chinese.
24. Zhao ZQ, Huang MH, Wan JQ, Su QP. Clinical analysis of eight cases of *Cryptosporidium* enteritis. J Fujian Med Coll. 1991;25(1):57. Chinese.
25. Xie WQ, Zheng ZZ, Zhao Y, Zhuo BY, Xiao YL. The first report of six cases of *Cryptosporidium* infection in Xiapu County in Fujian Province. Chin J Zoonoses. 1992;8(3):23–6. Chinese.
26. Zheng CR, Xiao ZL. Epidemiological investigation of Cryptosporidium infection and cryptosporidiosis in infants in Nanping of Fujian Province. Chin J Parasitic Dis Control. 1993;6(1):64. Chinese.
27. Su QP, Huang MH, Gao QF, Zhang JL, Wang BS, Zhang YS, et al. The first report of human cryptosporidiosisin Zhangzhou City of Fujian Province. Chin J Parasitol Parasitic Dis. 1994;12(1): 79–80. Chinese.
28. Chen H, Li CH, Xie ML, Wei XY, Yao LJ. The relationship between diarrhea and the infection of intestinal protozoa in Fuzhou district. Strait J Prev Med. 2002(6):14–6. Chinese.
29. Xu HZ, Lin GH, Meng JF, Huang ZM. Investigation of *Cryptosporidium* infection in diarrheal patients in Longhai City of Fujian Province. Chin J Zoonoses. 2005;21(4):282. Chinese.
30. Hong LX, Yang WC, Peng WF, Cui HJ. Survey on the *Cryptosporidium* and intestinal protozoa of man and animal. J Xiamen Univ. 1996;2:305–8. Chinese.
31. Zhao SY. Occurrence of cryptosporidiosis in humans in Kang County of Gansu Province. Gansu Sci Technol. 1996;12(1):25. Chinese.
32. Ling XM, Chen H, Yue W, Mao XR, Song JJ. Investigation of human *Cryptosporidium* infection in special populations. Chin J Parasitic Dis Control. 2001;14(3):1. Chinese.
33. Chen H, Mao XR, Ling XM, Song JJ. Epidemiological investigation of *Cryptosporidium* infection in diarrheal patients from three areas of Gansu Province. Chin J Parasitol Parasitic Dis. 2001;19(2):50. Chinese.
34. Ling XM, Chen H. Clinical observation of cryptosporidiosis with intestinal bacterial co–infection. J First Mil Meal Univ. 2005;7:919–20. Chinese.
35. Wang YN, Dong TT, Wang AX, Dang W, Lu ZL, Zhang S, et al. Epidemiological survey of *Cryptosporidium* and *Entamoeba histolytica* infection among patients with a chronic condition in Lanzhou. J Pathog Biol. 2013;8(10):934–6. Chinese.
36. Le XH, Wang H, Gou JZ, Chen XC, Yang GL, Yang QT, et al. Detection of *Cryptosporidium* infection among AIDS patients in Guangdong and Yunnan. Chin J Exp Clin Virol. 2008;22(5):339–41. Chinese.
37. Li WS, Shen SM. A preliminary report on the investigation of *Cryptosporidium* infection in humans and animals Guangdong Province. J First Mil Meal Univ. 1994;14(1):41. Chinese.
38. Zheng HB, Wang Y, Yao YX. Clinical Features of 4 Cases of infant *Cryptosporidium* enteritis. Acad J Guangzhou Med Coll. 2000;28(4):44–6. Chinese.
39. Zheng HB, Chen ZY, He CJ, Wang Y, Chen XW, Yao YX, et al. A study on the variations of causes, pathogens, and inducing factors for the pediatric nutritional dystrophy. J Mod Clin Med Bioeng. 2001;7(4):263–6. Chinese.
40. Cai XS, Lu DF, Chen SQ, Chen YJ, Wang T, Zheng DC. Etiological and clinical study of protracted and chronic diarrhea in children. Guangdong Med J. 2002;23(1):47–9. Chinese.
41. Li FW, Zhang XH, Wu FQ, Chen WN. Investigation of cryptosporidium infection in patients with chronic hepatitis b. New Med. 2007;38(2):81–3. Chinese.
42. Pang XL, Chen SY, Gao K, Mai HX, Han ZG, Xu HF, et al. Serum epidemiological analysis of opportunistic infection of pathogenic protozoa in HIV/AIDS. J Trop Med. 2015;15(10):1425–8. Chinese.
43. Wang L, Tian XH, He ZA, He CY, Ding Z, Xia YT, et al. *Cryptosporidium* infection of children in some rural areas of China. J Envir Hyg. 2015;5(4):328–30. Chinese.
44. Yao Y, Chen H, Liu X, Xiao N, Xiao Y, Huang Y, et al. Molecular epidemiological studies of cryptosporidiosis diarrhea in children of Guangzhou sentinel hospital. J Tro Medicine. 2014;14(1):60–3. Chinese.
45. Jiang JX, Ou WL, Lao HB. A case report of infantile cryptosporidiosis. Chin J Parasitol Parasitic Dis. 1995;13(2):77. Chinese.
46. Gan ZG, Chen JM, He XC, Luo J, Li YS, Pan QH, et al. Epidemiological survey of *Cryptosporidium* in humans and livestock in Liuzhou City of Guangxi. Chin J Parasitic Dis Control. 1995;8(1):20. Chinese.
47. Su H, Huang X, Qin Y, Qin S, Huang Z, Wu N, et al. An investigation on the gene types of *Cryptosporidium* among HIV/AIDS patients. J Med Pest Control. 2017;33(2):151–3. Chinese.
48. Wang ZF, Jiang ZH, Yu BX, Zhou DS, Lin Y, Tang WQ. Preliminary study on infection status and gene types of *Cryptosporidium* among HIV/AIDS patients in Guangxi. Chin J Schisto Control. 2016;28(5):550–3. Chinese.
49. Rong JQ, Wu GP, Chen LH, Yu YS, Qiu XZ. Epidemiological investigation of *Cryptosporidium* in villagers in Wengan County. J Qiannan Med Coll National. 1998;11(1):47–8. Chinese.
50. Rong JQ, Wu GP, Chen LH, Yu YS. Epidemiological survey of human cryptosporidiosis in Qiannan area. J Qiannan Med Coll National. 1999;1:52–4. Chinese.
51. Chen Y, Lang SY, Li JH, Qiu XL. Investigation of opportunistic parasitic infections in some populations in Guiyang area. J Guiyang Med Coll. 1999;24(3):243–4. Chinese.
52. Wang HY, Rong JQ, Wu GP. Investigation on cryptosporidiosis in some southern areas of Guizhou Province. J Trop MED. 2006;6:717–8. Chinese.
53. Miao ZF, Zhu X, Lv JP, Zhang WF, Zhang TS. Study on infantile infection with cryptosporidiosis. Chin J Pest Control. 1994;3:205–8. Chinese.
54. Miao ZF, Lv JP, Zhu X, Zhang TS, Shan LJ, Guo JS, et al. First report of infantile cryptosporidiosis in Hebei Province. Chin J Pest Control. 1994;3:210–1. Chinese.
55. Chen SL, Cao ZR, Li HL, Geng SJ. *Cryptosporidium* infection in diarrheal patients and mini-review. J Hebei Med Coll Contin Educ. 1996;3:18–9. Chinese.
56. Chen SL, Cao ZR, Li HL, Geng SJ. *Cryptosporidium* and unexplained diarrhea. Hebei Med J. 1997;19(5):288–90. Chinese.
57. Zhou YC, Chen SL, Cao ZR. *Cryptosporidium* infection in a patient with chronic diarrhea and detection of immune status. Lif Inf Pre Med. 1999;5(3):284. Chinese.
58. Wei YJ. Analysis of microbiological test results of acute diarrhea in infants. Guide China Med. 2014;12(10):127–8. Chinese.
59. Zhao XN, Zhang DM, Zhang LX, Liu XM. Investigation of *Cryptosporidium* infection in infants in Harbin area. Chin J Parasitic Dis Control. 1997;10(3):75. Chinese.
60. Niu Y, Li YH, Yu XH. Preliminary investigation of *Cryptosporidium* infection in diarrheal patients in Qiqihar area. J Qiqihar Med Coll. 2006, 27(2):184–5. Chinese.
61. Liu HX. Analysis of pathogenic microorganisms in fecal specimens of HIV patients with chronic diarrhea. J Practical Gynecol Endocrinol. 2017,4(15):64–6. Chinese.
62. Wang HZ, Jiao BX, Tian JH, Li M, Guo J, Liu Y, et al. Detection of *Cryptosporidium* infection among HIV/AIDS patients with chronic diarrhea in Beijing, Henan and Xinjiang of China. Chin J Epidemiol. 2011;32(9):927–9. Chinese.
63. Li SH, Zhang KR, Zhang QF, Ma JS. Investigation of intestinal protozoan infection in 388 patients with diarrhea. Chin J Parasitic Dis Control. 1992;5(2):158. Chinese.
64. Su YP, He LJ, Song JD, Lu LF, Zhang KR, Zhang QF. Investigation of *Cryptosporidium* infection in diarrhea infants in Henan Province. Henan J Prev Med. 1991;2(1):533–5. Chinese.
65. Su YP, Liu H, Yan XX, Sun QL, He ZL, Li GD, et al. Finding of cryptosporidiosis in humans in Zhoukou area of Henan Province. J Pract Parasitic Dis. 1994;2(3):41. Chinese.
66. Lu JZ, Su YP, He LJ, Li JA, Li XW, Ren YM. Three pediatric cases cryptosporidiosis in Wuzhi County of Henan Province. Chin J Parasitol Parasitic Dis. 1994;2:72. Chinese.
67. He LJ, Yan QY, Shi HF, Xue XL, Li W, Ma S, et al. Investigation on human intestinal parasite infections in Kaifeng of Henan Province. Henan Med Res. 1998;7(1):56–8. Chinese.
68. Wang Q, Zhou Y, Lv B, Fu KD, Chen L, Wang YQ, et al. Prevalence of intestinal parasite infection in Kaifeng City, Henan Province. J Trop Med. 2009;9(5):510–4. Chinese.
69. Wang RJ, Qi M, Zhao YF, Zhang XS, Ning CS, Zhang LX. Prevalence of Intestinal Parasitic Infection in Children in Linzhou, Henan Province. J Trop Med. 2009;9(10):1184–7. Chinese.
70. Li W, He LJ, Yan QY, Su YP, Zhao KY. Investigation on cryptosporidiosis in Henan. Strait J Prev Med. 2000;5:4–5. Chinese.
71. Xu LN, Wu GQ, Xi JW, Qi M, Yang N, Zhang LX, et al. Prevalence of intestinal parasitic infection in children in a hospitalized children in Zhengzhou. J Trop Med. 2011;11(1):17–8. Chinese.
72. Zhou YL, Wang YG, Li P, Liu JY, Wang HZ. Clinical study on 311 patients with AIDS related chronic diarrhea. Chin J Exp Clin Infect Dis. 2012;6(3):191–4. Chinese.
73. Wang L, Zhang H, Zhao X, Zhang L, Zhang G, Guo M, et al. Zoonotic *Cryptosporidium* species and *Enterocytozoon bieneusi* genotypes in HIV–positive patients on antiretroviral therapy. J Clin Microbiol. 2013;51(2):557–63.
74. Zhu MS, Song MH. *Cryptosporidium* infection in students of some primary and middle school in Shiyan City of Hubei Province. Chin J School Health. 2007;28(6):549. Chinese.
75. Zhu MS, Song MH. *Cryptosporidium* infection in kindergarten children in Shiyan City. Chin J School Health. 2007;28(11):1040. Chinese.
76. Zhu MS, Zhu J, Wang SJ, Song MH. A survey of *Cryptosporidium* infection among humans being in Shiyan, China. J Pathog Biol. 2009;4(9):685–6. Chinese.
77. Zhu J, Zhu MS, Wang SJ, Song MH. Investigation on the infection of *Cryptosporidium* among malignant tumor patients in Shiyan. J Trop Med. 2010,10(5):553–4. Chinese.
78. He ZA, Fu LX, Zhou WS, Chen J, Tian BQ, Wang L, et al. Investigation on *Cryptosporidium* infection of children in rural area of Chibi City, Hubei Province. J Public Health Prev Med. 2013;24(6):72–4. Chinese.
79. Min HL, Chen SM, Hu JX. Investigation on C*ryptosporidium* infection in children in Xianning area. J Public Health Prev Med. 2014;25(6):111–2. Chinese.
80. Liu X, Mao T, Wu P, Zhou R. Molecular epidemiology of *Cryptosporidium* infection in infants with diarrhea in Wuhan City. Chin J Schisto Control. 2017;29(2):188–91. Chinese.
81. Wang T, Fan Y, Koehler AV, Ma G, Li T, Hu M, et al. First survey of *Cryptosporidium*, *Giardia* and *Enterocytozoon* in diarrhoeic children from Wuhan, China. Infect Genet Evol. 2017;51:127–31.
82. Lu LA, Li CC, Fan ZZ, Chen YL. Fingding and epidemiological investigation of zoonotic cryptosporidiosis in humans and livestock in Hunan Province. Chin J Zoonoses. 1992;8(2):43–4. Chinese.
83. Zhang YY, Yu FY, Zhang XF, Zhang HY, An YQ. Investigation and clinical analysis of *Cryptosporidiu*m infection in diarrhea infants. J Appl Clin Pediatr. 1997;12(2):127. Chinese.
84. Huang MZ, Guan L, Zhou CX, Li DQ, Hu B. Infection of *Cryptosporidium* in child patients with diarhea in Changsha. Bull Hunan Med Univ. 1998;3:38–9. Chinese.
85. Huang MZ, Zhou CX, Guan L, Li DQ, Liu YY, Yu B, et al. Study of *Cryptosporidium* infection in adult patients with diarrhea. Curr Physician. 1998;3(10):42–3. Chinese.
86. Huang MZ, Guan L, Li DQ, Liu AZ, Dai WP, Chen X. Investigation of cryptosporidium infection in male intravenous drug users. Chin J Zoonoses. 2002;18(2):131. Chinese.
87. Huang MZ, Guan L, Xie MZ, Li DQ, Zhou J, Li ZY, et al. Study on condition of *Cryptosporidium* infection among male drug users in detoxification institute in Changsha City. Chin J Public Health. 2003;19(3):49–51. Chinese.
88. Zeng SH, Xiang YE, Yi SL. *Cryptosporidium* infection of human respiratory tract and exploration of examination methods. Pract Prev Med. 2003;10(1):103. Chinese.
89. Li HH, Li DQ, Huang MZ, Dai WP. Effects of nitric oxide on *Cryptosporidium* among intravenous drug abusers. Chin J Mod Med. 2003;21:11–3. Chinese.
90. Huang MZ, Li DQ, Guan L, Xie MZ, Liu H, Dai WP. Analysis of therapeutic effect on 151 cases of *Cryptosporidium* infection. Chin J Zoonoses. 2004;10:822. Chinese.
91. Li DQ, Li HH, Huang MZ, Dai WP. Study on the incidence of *Cryptosporidium* infection and the state of immune function of intravenous drug abusers. Chin J Zoonoses. 2005;2:153–5. Chinese.
92. Lu LA, Zhang WS, Li BB, Fan ZZ, Jiang CH. Etiological diagnosis and case-control study of cryptosporidiosis. Contemp Med. 2009,15(13):150–1. Chinese.
93. Xie RH, Chen GX, Ouyang SS. Analysis status of intestinal parasite infection among HIV/AIDS patients in Hengyang. Chin J Immun. 2015;31(5):695–7. Chinese.
94. Yu Z, Li F, Zeng Z, Huang Z, Fan Z, Jin Y, et al. Prevalence and clinical significance of *Cryptosporidium* infection in patients with hepatitis B virus–associated acute–on–chronic liver failure. Int J Infect Dis. 2011;15(12):e845–8.
95. Guan JS, Cui SL, Yan XL, Zhang JZ. Investigation of *Cryptosporidium* enteritis in Hohhot area. Acta Acad Med Nei Mongol. 1989;11(1):47–8. Chinese.
96. Xie YT, Wang L, Cui XC, Jin GC, Tong YF, Tian XH, et al. *Cryptosporidium* infection of children in pastoral areas of Inner Mongolian. J Envir Hyg. 2015;5(6):504. Chinese.
97. Han F, Xu S. Etiological diagnosis of cryptosporidiosis. Chin J Parasitol Parasitic Dis. 1989;1:3–5. Chinese.
98. Yao FB, Chen YG. Therapeutic effect of allicin in children with cryptosporidiosis. Acta Acad Med Xuzhou. 1989;1:56–7. Chinese.
99. Han F, Wang L, Wang RZ, Ge JJ, Shen JP. Investigation of cryptopsoridiosis in humans in Nanjing, China. Chin J Zoonoses. 1989;5(5):51. Chinese.
100. Ge JJ, Shen JP, Hu BY. Laboratory diagnosis of cryptosporidium enteritis. Shanghai J Med Lab Sci. 1990;5(2):65–6. Chinese.
101. Jiang Y, Ren J, Yuan Z, Liu A, Zhao H, Liu H, et al. *Cryptosporidium andersoni* as a novel predominant *Cryptosporidium* species in outpatients with diarrhea in Jiangsu Province, China. BMC Infect Dis. 2014;14:555.
102. Chen YG, Dai MX, Yao FB. Investigation on cryptosporidium infection in villagers of zhoupeng village, in Tongshan County. Acta Acad Med Xuzhou. 1990;1:40–2. Chinese.
103. Chen YG, Yao FB. A report of 14 cases of human cryptosporidiosis in Xuzhou area. Chin J Zoonoses. 1990;6(3):28. Chinese.
104. Tian SL, Zhang KY. An epidemiological survey of *Cryptosporidium* in children and a mini-review on research situation of human cryptosporidiosis in China. Chin J Public Health. 1991;7(4):169–71. Chinese.
105. Yao FB, Chen YG. *Cryptosporidium* enteritis in children: a clinical analysis of 66 cases. J Clin Pediatr. 1991;9(3):160–2. Chinese.
106. Chen YG, Li HS, Dai MX, Yao FB, Shi WS, Lu M. Epidemiological studies on human cryptosporidiosis in rural and urban areas of Jiangsu. Chin J Parasitol Parasitic Dis. 1993;3:49–52. Chinese.
107. Shen JP, Ge JJ, Hu BY. Pedigree investigation of cryptosporidium enteritis and mother-infant relationship. J Clin Pediatr. 1991;9(3):162–3. Chinese.
108. Shen JP, Ge JJ. Investigation and analysis of the infection rate of *Cryptosporidium* among 2018 children in kindergartens. J Pract Pediatr. 1991;6(3):132. Chinese.
109. Ge JJ, Shen JP, Cheng P, Chi FL. An epidemiological investigation of *Cryptosporidium* in fecal specimensfrom diarrheal patitens using modified Kinyoun acid fast staining method. J Clin Lab Sci. 1989;7(3):139. Chinese.
110. Sun X, Gu GM, Gu GS, Pan YX, Cao MX, Tang JL. Investigation of cryptosporidiosis in patients with diarrhea in Haian County. Chin J Public Health. 1992;8(8):350. Chinese.
111. Chen YG, Yao FB, Li HS, Shi WS, Dai MX, Lu M. *Cryptosporidium* infection and diarrhea in rural and urban areas of Jiangsu, People's Republic of China. J Clin Microbiol. 1992;30(2):492–4.
112. Zhang PR. Analysis of 10 cases of *Cryptosporidium* infection in children in Jinagsu Province. Clin Focus. 1998;13(22):1020–1. Chinese.
113. Zhu HS, Du XL, Yu RB, Xu JM, Zhu LF, Wu HW. Serum immunology investigation of *Cryptosporidium* infection among injection drug abusers. Chin J Schisto Control. 2008;5:364–6. Chinese.
114. Du XL, Ge JJ, Qin YF, Meng R, Liu Y, Chu K, et al. The epidemiological study on cryptosporidiosis in outpatients of Nanjing children's hospital. J Tro Medicine. 2009;9(4):382–5. Chinese.
115. Zheng H, Wang L, Zhang R, Ding Z. *Cryptosporidium* contamination in rural water supplies and *Cryptosporidium* infestation among children in Jiangsu Province. J Environ Hyg. 2015;5(5):418–21. Chinese.
116. Zheng H, He J, Wang L, Zhang R, Ding Z, Hu W. Risk factors and spatial clusters of *Cryptosporidium* infection among school–age children in a rural region of eastern China. Int J Environ Res Public Health. 2018;15(5). pii: E924.
117. Su SL, Chen GF, Wu GH, Huang AM. Investigation of cryptosporidiosis in children with diarrhea in Ganzhou City. J Gannan Med Coll. 1992;12(1):52. Chinese.
118. Xie ZJ, Zhang RQ, Huang WF, Liao YG, Su SL. Epidemiological and clinical study on intestinal parasites in adults with chronic diarrhea in Ganzhou area. J South Med Univ. 2008;28(6):1035–6. Chinese.
119. Guo J. Analysis of pathogen detection of stool samples of HIV infected patients complicated with chronic diarrhea. Chin Contin Med Educ. 2017;9(13):63–4. Chinese.
120. Song JP, Zhao JX, Gao H, Liu Y, Yue HX, Zhang J, et al. Serological detection of *Cryptosporidium* spp. infection in outpatients in Changchun. Chin J Parasitol Parasitic Dis. 2011;29(3):239–41. Chinese.
121. Li Y, Bing YY, Cong F, An CL. Investigation of *Cryptosporidium* infection in diarrheal patients and drug users in Shenyang area. Chin J Zoonoses. 2006;22(5):473–5. Chinese.
122. Chen XC, Su LY, Sai SY, Liu JH, Yang GL, Song JM. Investigation of human *Cryptosporidium* in south and central Shandong Province. J Taishan Med Coll. 1991;4:359–62. Chinese.
123. Gong YX, Cao SQ, Shi XZ, Zhou SC, Liu YX. Investigation of *Cryptosporidium* infection among children. Acta Acad Med Qingdao. 1992;28(2):144–6. Chinese.
124. Gong YX, Cao SQ, Shi XZ, Zhou SC. Investigation of *Cryptosporidium* infection in diarrheal patients in Qingdao area. Chin J Parasitic Dis Control. 1993;6(1):63. Chinese.
125. Yang GL, Song JM, Tian XL. Analysis of 33 cases of human cryptosporidiosis in Zoucheng City. Chin J Parasitic Dis Control. 1994;7(2):100. Chinese.
126. Gong YX, Cao SQ, Shi XZ, Han M, Zhou SC, Hao JH. Survey of human *Cryptosporidium* infection in Qingdao area. Acta Acad Med Qingdao. 1997;2:28–9. Chinese.
127. Cui W, Liang RW, Wang ZZ. Investigation of children *Cryptosporidium parvum* infection and epidemiology in Weifang area. Chin J Birth Health Hered. 2001;2:114–5+132. Chinese.
128. Cui W, Liang RW, Wang ZZ. The Investigation of children *Cryptosporidium parvum* infection and epidemiology in Weifang south mountains area. J Weifang Med Coll. 2001;1:11–2. Chinese.
129. Cui W, Liang RW, Wang ZZ. Epidemiological investigation of *Cryptosporidium* infection in children in Weifang area. Chin J Prev Med. 2001;35(4):73. Chinese.
130. Zhou YX. Investigation of *Cryptosporidium* infection in diarrheal patients in Heze City during the period of 2002-2003. Endemic Dis Bull. 2006;21(2):38. Chinese.
131. Xin L, Cui W, Liang R, Ji R, Sun X, Li R. Investigation on the infection of *Cryptosporidium* among malignant tumor patients. J Pathog Biol. 2007;2(4):307–8. Chinese.
132. Zhang Z, Li HX, Liu CH, Niu Q, Bai XL. An epidemiological survey of *Crptosporidium* in diarrheal patients as well as application of a recombinant cp23antigen in the detection of *C. parvum* in fecal specimens. Shandong Med J. 2009. 49(11):52–3. Chinese.
133. Su LY, Chen XC, Li YH, Liu JH. One case report of infantile cryptosporidiosis in Jinan area. Chin J Parasitic Dis Control. 1990;3(2):154. Chinese.
134. Zhou HF, Zhu M, Yuan JL, Xu F, Chen YH, Zhang ZX, et al. A survey on *Cryptosporidium parvum* cryptosporidiosis in different people of Luwan district Shanghai. Shanghai J Prev Med. 2005;17(9):430–2. Chinese.
135. Qian FX, Shi M, Shen SL, Liu YH, Gao T. Investigation of *Cryptosporidium* infection in diarrheal adults with or without HIV/AIDS. Chin J Misdiagn. 2011,11(3):752. Chinese.
136. Zhang XP, He YY, Wang ZY, Zhang YG, Zhu Q, Jiang SF, et al. Investigation on *Cryptosporidium* infections in diarrhea patients from the general hospitals in Shanghai. Chin Trop Med. 2016;16(12):1183–6. Chinese.
137. Feng Y, Wang L, Duan L, Gomez–Puerta LA, Zhang L, Zhao X, et al. Extended outbreak of cryptosporidiosis in a pediatric hospital, China. Emerg Infect Dis. 2012;18(2):312–4.
138. Chen S, Ai L, Tian L, Zhang Y, Tong X, Li H, et al. Investigation and fecal specimen detection of cryptozoite and other protozoon infection from patients with diarrhea. Chin J Zoonoses. 2012;28(8):815–9. Chinese.
139. Liu H, Shen Y, Yin J, Yuan Z, Jiang Y, Xu Y, et al. Prevalence and genetic characterization of *Cryptosporidium*, *Enterocytozoon*, *Giardia* and *Cyclospora* in diarrheal outpatients in China. BMC Infect Dis. 2014;14:25.
140. Hou YS, Li JQ, Fei ZD, Chen CH, Tian RH. Investigation of *Cryptosporidium* infection in children in Xi’ an area. Chin J Parasitic Dis Control. 1991;3:200. Chinese.
141. Fei ZD, Tian RH. A report of three cases of infantile cryptosporidiosis. Shaanxi Med J. 1992;7:52–3. Chinese.
142. Hou Q, Chen JM, Liu W. Infection and epidemiological characteristics of *Cryptosporidium* in outpatients in a children's hospital in Xi 'an. Contemp Med. 2011;17(24):53. Chinese.
143. Zhang YJ, Luo P, Gao R. Investigation of *Cryptosporidium* infection in children with diarrhea in a children's hospital in Chengdu City. J Practical Parasitic Dis. 2001;9(2):72–3. Chinese.
144. Su YQ. Detection and analysis of pathogenic microorganisms in fecal specimens of patients with HIV infection complicated with chronic diarrhea. Chin Health Ind. 2014;11(6):24–5. Chinese.
145. Yang Y, Zhou YB, Xiao PL, Shi Y, Chen Y, Liang S, et al. Prevalence of and risk factors associated with *Cryptosporidium* infection in an underdeveloped rural community of southwest China. Infect Dis Poverty. 2017 Jan 9;6(1):2.
146. Yang Y, Zhou Y, Cheng W, Pan X, Xiao P, Shi Y, et al. Prevalence and determinants of *Cryptosporidium* infection in an underdeveloped rural region of southwestern China. Am J Trop Med Hyg. 2017;96(3):595–601.
147. Hung CC, Tsaihong JC, Lee YT, Deng HY, Hsiao WH, Chang SY, et al. Prevalence of intestinal infection due to *Cryptosporidium* species among Taiwanese patients with human immunodeficiency virus infection. J Formos Med Assoc. 2007;106(1):31–5.
148. Zhang YR, Ji WH, Liu X, Yu AD, Zhu JR. Investigation of cryptosporidiosis in children in Tianjin. Chin J Parasitic Dis Control. 1993;4:317. Chinese.
149. Wang QJ, Zhang J. Investigation of *Cryptosporidium* infection in 1124 children in Tarim region, Xinjiang. Xinjiang Med J. 1995;3:187–8. Chinese.
150. Hong LX, Yang WC, Peng WF, Cui HJ. Survey on the *Cryptosporidium* and intestinal protozoa of man and animal. J Xiamen Univ. 1996;2:305–8. Chinese.
151. Zuo YX, Chen FQ, Fang L. Finding of human and calf cryptosporidiosis in Yunnan Province and experimental infections. Chin J Zoonoses. 1990;3:34–6. Chinese.
152. Fan B, He XY, Huang ZM, Wang WL, Ba WF, Su Q. Epidemiological investigation of cryptosporidiosis in Yuxi area. Chin J Pest Control. 1992;4:228–30. Chinese.
153. Fan B, He XY, Wang WL, Huang ZM, Su Q. Investigation of *Cryptosporidium* infection in Yuxi County, Yunnan Province. Chin J Parasitol Parasitic Dis. 1994;S1:265. Chinese.
154. Wang YK, Yu H, Yang JL, Yang HM, Zhang LL, Zhang L, et al. A survey of human intestinal protozoa in Yunnan. Chin J Parasitol Parasitic Dis. 1994(S1):99–102.
155. Zhang BX, Yu H, Tao H, Bai ZM, He YQ, Li YL et al. Investigation on intestinal protozoal infection in Yuanyang County, Yunnan Province. Chin J Parasitic Dis Control. 2002;6:22. Chinese.
156. Shen LJ, LiW. Investigation of *Cryptosporidium* infection in intravenous drug users in Dali. Chin J Public Health. 2005;11:21–2. Chinese.
157. Wang L, Pu D. Investigation of *Cryptosporidium* infection in AIDS patients in Kunming. J Pract Med Tech. 2011;18(4):360–1. Chinese.
158. Zhang BX, Yu H, Zhang LL, Tao H, Li YZ, Li Y, et al. Prevalence survey on *Cyclospora cayetanensis* and *Cryptosporidium* ssp. in diarrhea cases in Yunnan Province. Chin J Parasitol Parasitic Dis. 2002;20(2):106–8. Chinese.
159. Zhang BX, Yu H, Zhang LL, Tao H, Li YZ, Li Y, et al. Prevalence survey on *Cyclospora cayetanensis* and *Cryptosporidium* ssp. in diarrhea cases in Yunnan Province. Chin J Parasitol Parasitic Dis. 2002;20(2):106–8. Chinese.
160. Xing WL, Yang L, Liang SH, Liu QZ, Zheng XY. Investigation of *Cryptosporidium* infection in diarrheal patients in Wenzhou. J Wenzhou Med Coll. 1999;2:29–30. Chinese.
161. Lu SH ,Lin AF ,Chen R ,Wen LY, Cheng YZ, Chen XJ, et al. The diagnosis and analysis of *Cryptosporidium* infection in children of Zhejiang Province. Chin J Zoonoses. 2000;1:42–4. Chinese.
162. Cheng YZ, Chen XJ, Lin AF, Lu SH, Chen R, Wen LY, et al. A report of 26 cases of human cryptosporidiosis in Hangzhou area. Shanghai J Med Lab Sci. 2000;5:307. Chinese.
163. Wang L, Cui X, Tong Y, Jin G, Tian X, Xia Y, et al. Epidemiological investigation of *Cryptosporidium* in human and animal feces as well as study on the rapid method for detecting *Cryptosporidium*. J Pub Health Prev Med. 2015;26(4):26–8. Chinese.
164. Li FW, Xiang XD, Yu ZJ. A study on *Cryptosporidium* infection in patients with chronic severe hepatitis B. J Chin Physician. 2005;7(4):484-6. Chinese.
