# Supplementary material for: A retrospective epidemiological analysis of human Cryptosporidium infection in China during the past three decades (1987-2018)
Source: PLoS Negl Trop Dis. 2020 Mar 30;14(3):e0008146. doi: 10.1371/journal.pntd.0008146 (PMC7145189; doi:10.1371/journal.pntd.0008146)
Supplement: S2 Table — (DOCX) [file pntd.0008146.s003.docx]

S2 Table. Prevalence of *Cryptosporidium* in humans by year of publication in China.

| **Year** | **Examined no.** | **Positive no.** | **Prevalence (%)** | **No. of reports** | **Ref** |
| --- | --- | --- | --- | --- | --- |
| 1987 | 250 | 20 | 8.00 | 1 | [19] |
| 1989 | 7375 | 126 | 1.71 | 6 | [23,95,97–99,109] |
| 1990 | 5844 | 199 | 3.41 | 5 | [100,102,103,133,151] |
| 1991 | 19137 | 379 | 1.98 | 9 | [12,24,64,104,105,107,108,122,140] |
| 1992 | 15672 | 332 | 2.12 | 9 | [25,63,82,110,111,117,123,141,152] |
| 1993 | 12064 | 273 | 2.26 | 5 | [20,26,106,124,148] |
| 1994 | 12552 | 205 | 1.63 | 9 | [27,37,53,54,65,66,125,153,154] |
| 1995 | 1251 | 55 | 4.40 | 3 | [45,46,149] |
| 1996 | 391 | 10 | 2.56 | 4 | [30,31,55,150] |
| 1997 | 4985 | 129 | 2.59 | 4 | [56,59,83,126] |
| 1998 | 1881 | 42 | 2.23 | 5 | [49,67,84,85,112] |
| 1999 | 3225 | 104 | 3.22 | 4 | [50,51,57,160] |
| 2000 | 6125 | 176 | 2.87 | 5 | [21,38,70,161,162] |
| 2001 | 11911 | 291 | 2.44 | 8 | [1,32,33,39,127–129,143] |
| 2002 | 14831 | 180 | 1.21 | 7 | [9,28,40,86,155,158,159] |
| 2003 | 11925 | 350 | 2.94 | 6 | [2–4,87–89] |
| 2004 | 2996 | 221 | 7.38 | 3 | [5,6,90] |
| 2005 | 2365 | 218 | 9.22 | 6 | [29,34,91,134,156,164] |
| 2006 | 2689 | 79 | 2.94 | 5 | [18,52,60,121,130] |
| 2007 | 4099 | 213 | 5.20 | 5 | [41,74,75,131,147] |
| 2008 | 1394 | 537 | 38.52 | 3 | [36,113,118] |
| 2009 | 15105 | 198 | 1.31 | 6 | [68,69,76,92,114,132] |
| 2010 | 2561 | 136 | 5.31 | 2 | [15,77] |
| 2011 | 5593 | 194 | 3.47 | 7 | [62,71,94,120,135,142,157] |
| 2012 | 9532 | 273 | 2.86 | 8 | [10,11,13,16,22,72,137,138] |
| 2013 | 3436 | 104 | 3.03 | 4 | [7,35,73,78] |
| 2014 | 2076 | 146 | 7.03 | 6 | [44,58,79,101,139,144] |
| 2015 | 10445 | 359 | 3.44 | 9 | [8,14,17,42,43,93,96,115,163] |
| 2016 | 3252 | 39 | 1.20 | 2 | [48,136] |
| 2017 | 3455 | 327 | 9.46 | 7 | [47,61,80,81,119,145,146] |
| 2018 | 1637 | 18 | 1.10 | 1 | [116] |

Note: All the references in this table can be found in the reference list of S1 Table.
