# Supplementary material for: A retrospective epidemiological analysis of human Cryptosporidium infection in China during the past three decades (1987-2018)
Source: PLoS Negl Trop Dis. 2020 Mar 30;14(3):e0008146. doi: 10.1371/journal.pntd.0008146 (PMC7145189; doi:10.1371/journal.pntd.0008146)
Supplement: S3 Table — (DOCX) [file pntd.0008146.s004.docx]

S3 Table. Prevalence of *Cryptosporidium* by population in China.

| **Population** | **Clinical signs** | **Examined no** | **Positive no.** | **Prevalence (%)** | **Ref** |
| --- | --- | --- | --- | --- | --- |
| Children under 5 | Diarrhea | 203 | 7 | 3.45 | [1] |
|  |  | 385 | 31 | 8.05 | [26] |
|  |  | 271 | 4 | 1.48 | [38] |
|  |  | 54 | 1 | 1.85 | [45] |
|  |  | 107 | 2 | 1.87 | [54] |
|  |  | 931 | 13 | 1.40 | [59] |
|  |  | 1074 | 12 | 1.12 | [65] |
|  |  | 699 | 4 | 0.57 | [66] |
|  |  | 298 | 9^a^ | 3.02 | [80] |
|  |  | 200 | 3 | 1.50 | [141] |
|  | **Subtotal** | **4222** | **86** | **2.04** |  |
|  | NA | 889 | 28 | 3.15 | [9] |
|  |  | 120 | 16 | 13.33 | [23] |
|  |  | 136 | 12 | 8.82 | [50] |
|  |  | 137 | 12 | 8.76 | [52] |
|  |  | 4889 | 106 | 2.17 | [53] |
|  |  | 72 | 2 | 2.78 | [67] |
|  |  | 26 | 7 | 26.92 | [146] |
|  | **Subtotal** | **6269** | **183** | **2.92** |  |
| **Total** | | **10491** | **269** | **2.56** |  |
| Children over 5 or unspecific | Diarrhea | 3498 | 67 | 1.92 | [12] |
|  |  | 830 | 34 | 4.10 | [20] |
|  |  | 649 | 46 | 7.09 | [21] |
|  |  | 62 | 8 | 12.90 | [24] |
|  |  | 42 | 4 | 9.52 | [39] |
|  |  | 693 | 16 | 2.31 | [40] |
|  |  | 348 | 12^a^ | 3.45 | [44] |
|  |  | 483 | 12 | 2.48 | [64] |
|  |  | 500 | 10^a^ | 2.00 | [81] |
|  |  | 250 | 20 | 8.00 | [83] |
|  |  | 102 | 5 | 4.90 | [84] |
|  |  | 140 | 2^a^ | 1.43 | [94] |
|  |  | 140 | 5 | 3.57 | [95] |
|  |  | 478 | 20 | 4.18 | [98] |
|  |  | 3604 | 49 | 1.36 | [99] |
|  |  | 6498 | 163 | 2.51 | [107] |
|  |  | 1000 | 13 | 1.30 | [109] |
|  |  | 480 | 10 | 2.08 | [112] |
|  |  | 121 | 2 | 1.65 | [117] |
|  |  | 190 | 3 | 1.58 | [140] |
|  |  | 462 | 36 | 7.79 | [142] |
|  |  | 406 | 12 | 2.96 | [143] |
|  |  | 1200 | 11 | 0.92 | [148] |
|  |  | 962 | 59 | 6.13 | [160] |
|  |  | 548 | 57 | 10.40 | [161] |
|  |  | 300 | 26 | 8.67 | [162] |
|  |  | 140 | 2 | 1.43 | [164] |
|  | **Subtotal** | **24126** | **704** | **2.92** |  |
|  | Non-gastrointestinal illnesses | 6284 | 102^a^ | 1.62 | [137] |
|  | NA | 1204 | 42 | 3.49 | [5] |
|  |  | 889 | 28 | 3.15 | [6] |
|  |  | 192 | 7 | 3.65 | [8] |
|  |  | 1098 | 9 | 0.82 | [9] |
|  |  | 102 | 2 | 1.96 | [10] |
|  |  | 388 | 16 | 4.12 | [22] |
|  |  | 112 | 3 | 2.68 | [37] |
|  |  | 5458 | 141 | 2.58 | [43] |
|  |  | 181 | 18 | 9.94 | [49] |
|  |  | 378 | 11 | 2.91 | [50] |
|  |  | 387 | 11 | 2.84 | [52] |
|  |  | 356 | 0 | 0 | [67] |
|  |  | 434 | 3 | 0.69 | [68] |
|  |  | 1949 | 3 | 0.15 | [69] |
|  |  | 1996 | 2 | 0.10 | [71] |
|  |  | 941 | 62 | 6.59 | [74] |
|  |  | 1118 | 51 | 4.56 | [75] |
|  |  | 1836 | 110 | 5.99 | [76] |
|  |  | 1128 | 36 | 3.19 | [78] |
|  |  | 1035 | 58 | 5.60 | [79] |
|  |  | 2262 | 57 | 2.52 | [82] |
|  |  | 1002 | 42 | 4.19 | [96] |
|  |  | 618 | 60 | 9.71 | [100] |
|  |  | 215 | 11 | 5.12 | [102] |
|  |  | 1065 | 1 | 0.09 | [104] |
|  |  | 2549 | 86 | 3.37 | [105] |
|  |  | 6145 | 127 | 2.07 | [106] |
|  |  | 2018 | 16 | 0.79 | [108] |
|  |  | 5089 | 89 | 1.75 | [111] |
|  |  | 2268 | 22 | 0.97 | [114] |
|  |  | 1651 | 18 | 1.09 | [115] |
|  |  | 1637 | 18 | 1.10 | [116] |
|  |  | 1521 | 14 | 0.92 | [122] |
|  |  | 969 | 26 | 2.68 | [123] |
|  |  | 2206 | 68 | 3.08 | [127] |
|  |  | 1943 | 55 | 2.83 | [128] |
|  |  | 3729 | 75 | 2.01 | [129] |
|  |  | 305 | 37 | 12.13 | [146] |
|  |  | 1124 | 53 | 4.72 | [149] |
|  |  | 114 | 2 | 1.75 | [150] |
|  |  | 796 | 17 | 2.14 | [153] |
|  | **Subtotal** | **60408** | **1507** | **2.49** |  |
| **Total** | | **90818** | **2313** | **2.55** |  |
| Teenagers | NA | 149 | 3 | 2.01 | [10] |
|  |  | 40 | 2 | 5.00 | [49] |
|  |  | 383 | 8 | 2.09 | [50] |
|  |  | 289 | 8 | 2.77 | [52] |
|  |  | 90 | 0 | 0 | [67] |
|  |  | 380 | 0 | 0 | [68] |
|  |  | 503 | 10 | 1.99 | [82] |
|  |  | 340 | 49 | 14.41 | [146] |
| **Total** | | **2174** | **80** | **3.68** |  |
| Adults | NA | 443 | 8 | 1.81 | [8] |
|  |  | 3120 | 26 | 0.83 | [9] |
|  |  | 469 | 27 | 5.76 | [10] |
|  |  | 850 | 26 | 3.06 | [22] |
|  |  | 41 | 0 | 0 | [23] |
|  |  | 70 | 2 | 2.86 | [37] |
|  |  | 114 | 3 | 2.63 | [49] |
|  |  | 1049 | 10 | 0.95 | [50] |
|  |  | 926 | 9 | 0.97 | [52] |
|  |  | 394 | 0 | 0.00 | [67] |
|  |  | 5279 | 3 | 0.06 | [68] |
|  |  | 713 | 9 | 1.26 | [76] |
|  |  | 974 | 2 | 0.21 | [82] |
|  |  | 15 | 1 | 6.67 | [102] |
|  |  | 939 | 8 | 0.85 | [105] |
|  |  | 1248 | 9 | 0.72 | [106] |
|  |  | 384 | 113^b^ | 29.43 | [113] |
|  |  | 314 | 1 | 0.32 | [122] |
|  |  | 1356 | 16 | 1.18 | [127] |
|  |  | 964 | 113 | 11.72 | [146] |
|  |  | 1654 | 16 | 0.97 | [153] |
| **Total** | | **21316** | **402** | **1.89** |  |
| Patients | Diarrhea | 827 | 46 | 5.56 | [2] |
|  |  | 278 | 8 | 2.88 | [9] |
|  |  | 592 | 5^c^ | 0.84 | [14] |
|  |  | 2344 | 30^b^ | 1.28 | [15] |
|  |  | 210 | 8 | 3.81 | [27] |
|  |  | 3116 | 20 | 0.64 | [28] |
|  |  | 248 | 7 | 2.82 | [29] |
|  |  | 186 | 13 | 6.99 | [32] |
|  |  | 1840 | 41 | 2.23 | [33] |
|  |  | 580 | 54 | 9.31 | [34] |
|  |  | 73 | 1 | 1.37 | [46] |
|  |  | 150 | 1 | 0.67 | [51] |
|  |  | 49 | 3 | 6.12 | [55] |
|  |  | 149 | 9 | 6.04 | [56] |
|  |  | 69 | 2 | 2.90 | [57] |
|  |  | 330 | 11 | 3.33 | [60] |
|  |  | 388 | 10 | 2.58 | [63] |
|  |  | 52 | 2 | 3.85 | [85] |
|  |  | 1014 | 13 | 1.28 | [97] |
|  |  | 978 | 10 | 1.02 | [99] |
|  |  | 232 | 23^a^ | 9.91 | [101] |
|  |  | 314 | 1 | 0.32 | [110] |
|  |  | 210 | 4 | 1.90 | [118] |
|  |  | 283 | 9 | 3.18 | [121] |
|  |  | 2256 | 61 | 2.70 | [124] |
|  |  | 237 | 6 | 2.53 | [130] |
|  |  | 112 | 7 | 6.25 | [132] |
|  |  | 23 | 1 | 4.35 | [133] |
|  |  | 393 | 29 | 7.38 | [135] |
|  |  | 2817 | 37 | 1.31 | [136] |
|  |  | 109 | 10^a^ | 9.17 | [138] |
|  |  | 252 | 34^a^ | 13.49 | [139] |
|  |  | 73 | 9 | 12.33 | [151] |
|  |  | 1640 | 84 | 5.12 | [152] |
|  |  | 378 | 20 | 5.29 | [158] |
|  |  | 98 | 1 | 1.02 | [160] |
|  | **Subtotal** | **22900** | **630** | **2.75** |  |
|  | HBV+ | 556 | 19 | 3.42 | [41] |
|  |  | 340 | 14^a^ | 4.12 | [94] |
|  |  | 51 | 11 | 21.57 | [145] |
|  |  | 218 | 13 | 5.96 | [164] |
|  | **Subtotal** | **1165** | **57** | **4.89** |  |
|  | Cancer | 60 | 6 | 10.00 | [35] |
|  |  | 217 | 106 | 48.85 | [77] |
|  |  | 108 | 72 | 66.67 | [131] |
|  | **Subtotal** | **385** | **184** | **47.79** |  |
|  | NA and others (diabetic, hypertension and respiratory diseases) | 100 | 7 | 7.00 | [35] |
|  |  | 100 | 3 | 3.00 | [35] |
|  |  | 356 | 3 | 0.84 | [88] |
|  |  | 36 | 3 | 8.33 | [9] |
|  |  | 2134 | 41 | 1.92 | [92] |
|  |  | 4506 | 103 | 2.29 | [100] |
|  |  | 2046 | 65^b^ | 3.18 | [120] |
|  |  | 2853 | 42 | 1.47 | [152] |
|  |  | 340 | 9 | 2.65 | [35] |
|  | **Subtotal** | **12471** | **276** | **2.21** |  |
| HIV-positive patients | Diarrhea | 67 | 4 | 5.97 | [16] |
|  |  | 125 | 11 | 8.80 | [17] |
|  |  | 30 | 7^a^ | 23.33 | [18] |
|  |  | 58 | 9 | 15.52 | [61] |
|  |  | 186 | 28 | 15.05 | [62] |
|  |  | 207 | 28 | 13.53 | [72] |
|  |  | 91 | 13 | 14.29 | [119] |
|  |  | 79 | 13 | 16.46 | [144] |
|  | **Subtotal** | **843** | **113** | **13.40** |  |
|  | NA | 342 | 32 | 9.36 | [7] |
|  |  | 302 | 21 | 6.95 | [11] |
|  |  | 302 | 25 | 8.28 | [13] |
|  |  | 212 | 9 | 4.25 | [36] |
|  |  | 450 | 78^b^ | 17.33 | [42] |
|  |  | 258 | 6^a^ | 2.33 | [47] |
|  |  | 285 | 2 | 0.70 | [48] |
|  |  | 683 | 10^a^ | 1.46 | [73] |
|  |  | 152 | 20 | 13.16 | [93] |
|  |  | 14 | 2 | 14.29 | [145] |
|  |  | 1376 | 9^a^ | 0.65 | [147] |
|  |  | 30 | 18 | 60.00 | [157] |
|  | **Subtotal** | **4406** | **232** | **5.27** |  |
| **Total** |  | **5249** | **345** | **6.57** |  |
| HIV-negative patients | NA | 303 | 9 | 2.97 | [13] |
|  |  | 180 | 20^b^ | 11.11 | [42] |
|  |  | 150 | 0 | 0 | [48] |
|  |  | 683 | 1^a^ | 0.15 | [73] |
| **Total** |  | **1316** | **30** | **2.28** |  |
| Drug users | NA | 294 | 27 | 9.18 | [86] |
|  |  | 903 | 172 | 19.05 | [87] |
|  |  | 370 | 34 | 9.19 | [89] |
|  |  | 903 | 151 | 16.72 | [90] |
|  |  | 179 | 34 | 18.99 | [91] |
|  |  | 588 | 411^b^ | 69.90 | [113] |
|  |  | 70 | 6 | 8.57 | [121] |
|  |  | 500 | 84 | 16.80 | [156] |
| **Total** | | **3807** | **919** | **24.14** |  |

Note: *Cryptosporidium* infection or cryptosporidiosis was identified based on microscopic examination after staining in most epidemiological studies except those by PCR and sequencing ^a^, by immunological methods ^b^ and by xTAG GPP multiplex PCR ^c^

All the references in this table can be found in the reference list of S1 Table.
