# Supplementary material for: A retrospective epidemiological analysis of human Cryptosporidium infection in China during the past three decades (1987-2018)
Source: PLoS Negl Trop Dis. 2020 Mar 30;14(3):e0008146. doi: 10.1371/journal.pntd.0008146 (PMC7145189; doi:10.1371/journal.pntd.0008146)
Supplement: S4 Table — (DOCX) [file pntd.0008146.s005.docx]

S4 Table. Prevalence of *Cryptosporidium* in humans by living environment (rural/urban areas) in China.

| **Rural areas** | | | **Urban areas** | | | **Ref** |
| --- | --- | --- | --- | --- | --- | --- |
| **Examined no** | **Positive no.** | **Prevalence (%)** | **Examined no** | **Positive no.** | **Prevalence (%)** |  |
| 2145 | 38 | 1.77 | 3276 | 36 | 1.10 | [9] |
| 2308 | 41 | 1.78 | 1740 | 13 | 0.75 | [3] |
| 1599 | 40 | 2.50 | 347 | 1 | 0.29 | [50] |
| 421 | 11 | 2.61 | 510 | 2 | 0.39 | [59] |
| 1943 | 55 | 2.83 | 1786 | 20 | 1.12 | [129] |
| 680 | 26 | 3.82 | 289 | 0 | 0 | [123] |
| 561 | 23 | 4.10 | 5937 | 140 | 2.36 | [107] |
| 520 | 27 | 5.19 | 684 | 15 | 2.19 | [5] |
| 2066 | 117 | 5.66 | 483 | 2 | 0.41 | [76] |
| 400 | 36 | 9.00 | 427 | 10 | 2.34 | [2] |
| 419 | 39 | 9.31 | 699 | 12 | 1.72 | [75] |
| 513 | 49 | 9.55 | 547 | 11 | 2.01 | [160] |
| 87 | 9 | 10.34 | 189 | 7 | 3.70 | [41] |
| 74 | 8 | 10.81 | 144 | 5 | 3.47 | [164] |
| 373 | 48 | 12.87 | 568 | 14 | 2.46 | [74] |

Note: All references in this table can be found in the reference list of S1 Table.
