# Supplementary material for: A retrospective epidemiological analysis of human Cryptosporidium infection in China during the past three decades (1987-2018)
Source: PLoS Negl Trop Dis. 2020 Mar 30;14(3):e0008146. doi: 10.1371/journal.pntd.0008146 (PMC7145189; doi:10.1371/journal.pntd.0008146)
Supplement: S5 Table — (DOCX) [file pntd.0008146.s006.docx]

S5 Table. Prevalence of *Cryptosporidium* in humans by season in China.

| **Province (Abbreviation)^a^** | **Positive no./ Examined no (%)** | | | | **Ref** |
| --- | --- | --- | --- | --- | --- |
|  | **spring** | **summer** | **autumn** | **winter** |  |
| Jiangsu (JS) | 1/32 (3.13) | 5/99 (5.05) | 16/55 (29.09) | 1/46 (2.17) | [101] |
| Fujian (FJ) | 3/885 (0.34) | 10/713 (1.40) | 4/932 (0.43) | 3/586 (0.51) | [28] |
| Hunan (HN) | 6/482 (1.24) | 33/1984 (1.66) | 29/910 (3.19) | 1/363 (0.28) | [82] |
| Shanghai (SH) | 3/345 (0.87) | 26/1301 (2.00) | 7/770 (0.91) | 1/401 (0.25) | [136] |
| Shandong (SD) | 9/461 (1.95) | 19/538 (3.53) | 24/539 (4.45) | 3/405 (0.74) | [128] |

Note: All the references in this table can be found in the reference list of S1 Table. In China, spring begins in March, summer in June, autumn in September, and winter in December.
