# Supplementary material for: A retrospective epidemiological analysis of human Cryptosporidium infection in China during the past three decades (1987-2018)
Source: PLoS Negl Trop Dis. 2020 Mar 30;14(3):e0008146. doi: 10.1371/journal.pntd.0008146 (PMC7145189; doi:10.1371/journal.pntd.0008146)
Supplement: S6 Table — (DOCX) [file pntd.0008146.s007.docx]

S6 Table. Drugs, dosages and negative conversion ratios of oocysts in fecal specimens under microscopy.

| **Group** | **Drug** | **Dosage** | **Case number** | **Negative conversion ratio (%)** | | | | **Ref** |
| --- | --- | --- | --- | --- | --- | --- | --- | --- |
|  |  |  |  | **1-7d** | **8-14d** | **>14d** | **Total** |  |
| **Diarrheal Children** | **Allicin** | NA | 12 | 91.7 | 8.3 |  | 100 | [23] |
|  |  | NA | 13 |  |  |  | 100 | [59] |
|  |  | NA | 6 |  |  |  | 100 | [121] |
|  |  | NA | 12^a^ |  |  |  | 100 | [123] |
|  |  | 10-20 mg/time (the initial dose is 20-40 mg), four times daily | 5 | 80 | 20 |  | 100 | [64] |
|  |  | 20-40 mg/time, four times daily | 5 |  |  |  | 100 | [65] |
|  |  | aged < 1years: 1 capsule three times daily; aged 1-5 years: two capsule three times daily; aged > 5years: three capsule three times daily | 43 | 16.3 | 18.6 | 60.4 | 95.3 | [105] |
|  | **Allicin+antibiotics** |  |  |  |  |  |  |  |
|  | allicin+ amikacin + ampicillin | NA | 4 |  |  |  | 100 | [38] |
|  | allicin +gentamicin + pipemidic acid | NA | 16 | 100 |  |  | 100 | [53] |
|  | allicin +metronidazole | NA | 17 | 100 |  |  | 100 |  |
|  | allicin +spiramycin | NA | 33 | 100 |  |  | 100 |  |
|  | allicin + sulfamethoxazole | NA | 20 | 100 |  |  | 100 |  |
|  | allicin +metronidazole + spiramycin | NA | 1 | 100 |  |  | 100 |  |
|  | **Antibiotics** |  |  |  |  |  |  |  |
|  | ampicillin, gentamicin, pipemidic acid and norfloxacin used interchangeably | NA | 8 |  |  |  | 100 | [27] |
|  | metronidazole + spiramycin | NA | 19 | 100 |  |  | 100 | [53] |
|  | spiramycin | 80 mg/kg/d | 3 | 100 |  |  | 100 | [140] |
|  | spiramycin or pipemidic acid + spiramycin | NA | 3 |  | 100 |  | 100 | [141] |
|  | spiramycin | 0.3 g/kg/d in three divided doses | 8 | 87.5 | 12.5 |  | 100 | [160] |
|  | **Others** |  |  |  |  |  |  |  |
|  | berberine + gentamicin | NA | 5 |  |  |  | 100 | [95] |
|  | gentamicin +lactasin + B complex vitamin solution | NA | 1 |  | 100 |  | 100 | [110] |
|  | imodium | 1 capsule 3 times daily | 4 | 100 |  |  | 100 | [65] |
| **Diarrheal Adults** | **Allicin** | NA | 3 |  |  |  | 100 | [121] |
| **Diarrheal patients without specific ages** | **Allicin** | NA | 1 |  |  |  | 100 | [51] |
|  |  | 20-40 mg/time, three times daily | 30 | 96.7 | 3.3 |  | 100 | [70] |
|  |  | NA | 37 |  |  |  | 94.6 | [99] |
|  |  |  |  |  |  |  |  |  |
|  |  | NA | 20 |  |  |  | 100 | [124] |
|  |  | children: one capsule four times daily (the initial dose is two capsules); double dose for adults | 26 | 92.3 | 7.7 |  | 100 | [126] |
|  | **Others** |  |  |  |  |  |  |  |
|  | trimethoprim-sulfamethoxazole (TMP-SMZ) + multienzyme tablets | TMP-SMZ, 0.5g/time (the initial dose is 1 g, children can cut down according to the circumstance), two times daily + multienzyme tablets, 2-3 tablets 3 times daily | 13 | 84.6 | 15.4 |  | 100 | [70] |
| **Drug users** | **Allicin** | 40mg/time, four times daily | 36 |  | 72.2 |  | 72.2 | [90] |
|  |  | NA | 6 |  |  |  | 100 | 121] |
|  | **Allicin+** **acetylspiramycin** | acetylspiramycin, 0.2g/time, four times daily + allicin, 40mg/time, four times daily | 38 |  | 92.1 |  | 92.1 | [90] |
|  | **Antibiotics** |  |  |  |  |  |  |  |
|  | acetylspiramycin | 0.2 g/time, three times daily | 27 | 85 | 4.0 | 11.0 | 100 | [86] |
|  |  | 0.2g/time, four times daily | 43 |  | 76.7 |  | 76.7 | [90] |

Note: All the references in this table can be found in the reference list of S1 Table. Negative conversion ratios of three case-control studies between untreated and treated groups: 61.8% (21/34) versus 80.3% (94/117) [90]; 100% (6/6) versus 94.6% (35/37) [99]; 86.4% (19/22) versus 100% (20/20) [124].

^a^ 12 patients showed at least one gastrointestinal symptom (abdominal pain, abdominal distension, diarrhea, nausea and vomiting).
